# Seasonality and longer-term development generate temporal dynamics in the *Populus* microbiome

**DOI:** 10.1128/msystems.00886-23

**Published:** 2024-02-29

**Authors:** William A. Argiroff, Alyssa A. Carrell, Dawn M. Klingeman, Nicholas C. Dove, Wellington Muchero, Allison M. Veach, Toni Wahl, Steven J. Lebreux, Amber B. Webb, Kellie Peyton, Christopher W. Schadt, Melissa A. Cregger

**Affiliations:** 1 Biosciences Division, Oak Ridge National Laboratory, Oak Ridge, Tennessee, USA; 2 Department of Integrative Biology, The University of Texas, San Antonio, Texas, USA; 3 Department of Microbiology, University of Tennessee, Knoxville, Tennessee, USA; University of Dundee, Dundee, United Kingdom

**Keywords:** phyllosphere, fine roots, rhizosphere, temporal patterns, microbial community assembly

## Abstract

Temporal variation in community composition is central to our understanding of the assembly and functioning of microbial communities, yet the controls over temporal dynamics for microbiomes of long-lived plants, such as trees, remain unclear. Temporal variation in tree microbiomes could arise primarily from seasonal (i.e., intra-annual) fluctuations in community composition or from longer-term changes across years as host plants age. To test these alternatives, we experimentally isolated temporal variation in plant microbiome composition using a common garden and clonally propagated plants, and we used amplicon sequencing to characterize bacterial/archaeal and fungal communities in the leaf endosphere, root endosphere, and rhizosphere of two *Populus* spp. over four seasons across two consecutive years. Microbial community composition differed among seasons and years (which accounted for up to 21% of the variation in microbial community composition) and was correlated with seasonal dissimilarity in climatic conditions. However, microbial community dissimilarity was also positively correlated with time, reflecting longer-term compositional shifts as host trees aged. Together, our findings demonstrate that temporal patterns in tree microbiomes arise from both seasonal fluctuations and longer-term changes, which interact to generate unique seasonal patterns each year. In addition to shedding light on two important controls over the assembly of plant microbiomes, our results also suggest future studies of tree microbiomes should account for background temporal dynamics when testing the drivers of spatial patterns in microbial community composition and temporal responses of plant microbiomes to environmental change.

**IMPORTANCE:**

Microbiomes are integral to the health of host plants, but we have a limited understanding of the factors that control how the composition of plant microbiomes changes over time. Especially little is known about the microbiome of long-lived trees, relative to annual and non-woody plants. We tested how tree microbiomes changed between seasons and years in poplar (genus *Populus*), which are widespread and ecologically important tree species that also serve as important biofuel feedstocks. We found the composition of bacterial, archaeal, and fungal communities differed among seasons, but these seasonal differences depended on year. This dependence was driven by longer-term changes in microbial composition as host trees developed across consecutive years. Our findings suggest that temporal variation in tree microbiomes is driven by both seasonal fluctuations and longer-term (i.e., multiyear) development.

## INTRODUCTION

Temporal patterns in community assembly are central to our understanding of the maintenance of biodiversity (1, 2), ecosystem functioning (3), and community responses to environmental change (4, 5). While the temporal variability of plant and animal communities has long been a focus of ecological research, a growing number of studies have also investigated temporal dynamics in microbial communities, such as those inhabiting the mammalian gut (6–8), soil (9–11), and aquatic ecosystems (12–14). However, less is known about temporal variation of microbial communities that associate directly with plants (15). Given the central role of plant microbiomes in mediating host growth, pathogen resistance, and responses to ongoing environmental change (16–18), identifying the mechanisms that control the temporal assembly of plant microbiomes has important implications for our ability to predict, maintain, and enhance future ecosystem functioning and services.

Seasonal fluctuations in community composition may be an important component of temporal variation in the plant microbiome (15), but our understanding of these dynamics is limited primarily to annual and non-woody plant species. Seasonal changes in the composition of plant microbiomes likely arise from seasonal variation in climatic conditions and resulting changes in host physiology (19, 20), which could generate predictable shifts in microbial community composition that depend on time of year. For example, the relative abundance of specific microbial groups in the phyllosphere of switchgrass and miscanthus depends on season (21–23), suggesting there are interannually consistent seasonal patterns in the microbiomes of these perennial crops. Numerous other studies have revealed changes in the microbiomes of a variety of annual and perennial plant species throughout single growing seasons (24–26), which could reflect seasonal shifts in microbial community composition. However, relatively little is known about microbial communities associated with long-lived trees (27), and it therefore remains unclear whether seasonal dynamics are important contributions to temporal variation in their composition.

Temporal patterns in tree microbiomes could instead be driven by longer-term shifts in community composition. Plants at different stages of development tend to harbor distinct microbial communities (28–30). Accordingly, the composition of tree-associated microbial communities may vary with tree age at decadal time scales, possibly due to metabolic changes occurring throughout host development that subsequently structure microbial communities (31–33) or microbial community succession that corresponds to time since germination (34). This evidence presents the possibility that tree microbiomes change continuously over time, causing seasonal fluctuations and longer-term shifts to occur simultaneously. However, studies of intra-annual variation in the composition of tree microbiomes typically span a single year (20, 35–37). Consequently, it has remained challenging to disentangle seasonal variation from longer-term shifts in tree-associated microbial communities, as well as from numerous climatic, edaphic, and host genotypic differences that correlate with spatial variation in the composition of plant microbiomes (37–42).

Here, we evaluated whether seasonality or longer-term changes in microbial community composition drive temporal dynamics of the *Populus* microbiome. The tree genus *Populus* includes numerous widespread and ecologically important riparian and early-successional species (43), which are potentially valuable biofuel feedstocks (44) and ideal model trees for the study of plant-microbe interactions due to their experimental tractability and well-characterized genetics (27). We grew 10 genotypes of two *Populus* spp. (*Populus deltoides* and *Populus trichocarpa*) in a common garden to isolate temporal assembly patterns in the *Populus* microbiome by experimentally controlling for the effects of spatial variation in the environment and host genotype on microbial community composition. We used amplicon sequencing to characterize intra-annual and interannual changes in bacterial, archaeal, and fungal communities across four seasons over 2 consecutive years, and we used these data to test two competing hypotheses. First, if seasonality dominates temporal variation in the *Populus* microbiome, we predicted that microbial community composition should fluctuate consistently between seasons across years and be correlated with seasonal variation in environmental conditions. Alternatively, if longer-term changes are the primary driver of temporal variation, microbial community composition should instead be correlated with time. Because microbial community composition and function differ among plant-associated habitats (38, 45), we separately characterized microbial communities inhabiting leaves, fine roots, and rhizosphere soils to understand whether temporal patterns differed across these host-associated habitats. Furthermore, *Populus* microbiomes often differ among host genotypes (38, 39), but it is unclear if temporal patterns also depend on genotype. Thus, an additional objective of our work was to understand if temporal patterns in microbial community composition were generalizable across *Populus* spp. Finally, we evaluated which microbial groups drove temporal dynamics in the *Populus* microbiome to understand potential consequences for community functioning.

## RESULTS

### Seasonal patterns in the *Populus* microbiome

**Fig 1:**
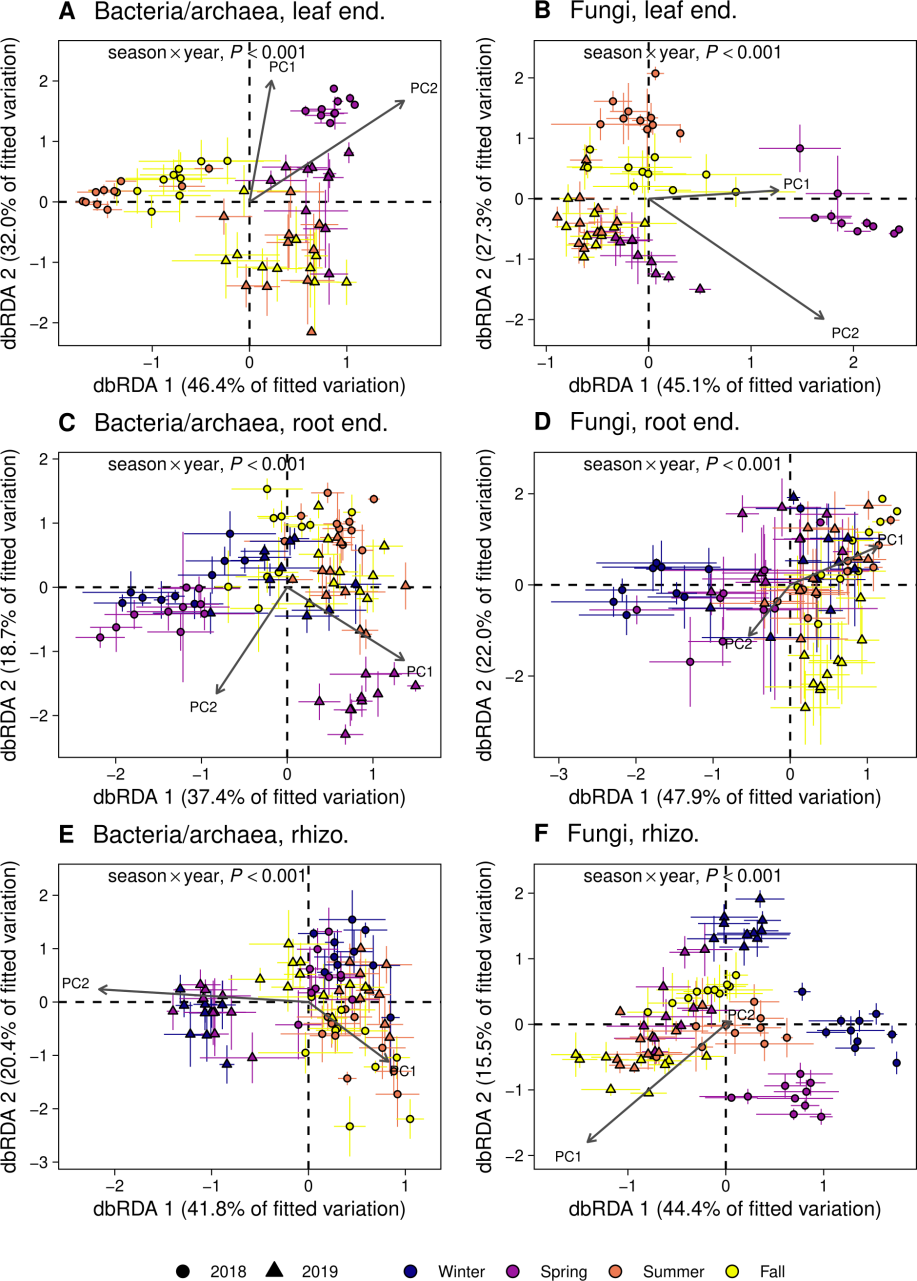
Ordinations developed from distance-based redundancy analysis (dbRDA) using Bray-Curtis dissimilarity calculated from subsampled ASV tables. dbRDA was performed on six separate ASV tables representing bacterial/archaeal (**A**) and fungal (**B**) communities in the leaf endosphere, bacterial/archaeal (**C**) and fungal (**D**) communities in the root endosphere, and bacterial/archaeal (**E**) and fungal (**F**) communities in rhizosphere soil. *P* values corresponding to the interaction between season and year as predictors of microbial community composition were determined from permutational multivariate analysis of variance (PERMANOVA; Table S1). Among bacterial and archaeal communities, temporal variables (season, year, and their interaction) accounted for 21% of variation in the leaf endosphere, 9% in the root endosphere, and 12% in the rhizosphere, whereas temporal variables explained 21%, 8%, and 19% for fungal communities in these habitats (PERMANOVA; Table S1). Season, year, host species, environmental PC1 (Fig. S1B), and environmental PC2 (Fig. S1B) were included as constraining variables in dbRDA. Maximum daily temperature, minimum daily temperature, vapor pressure, and day length increased with increasing values of PC1, whereas increasing values of PC2 corresponded to increasing short-wave radiation and decreasing precipitation (Fig. S1B). Microbial community composition was significantly correlated with all constraining variables for each community (bacteria/archaea and fungi) in each plant-associated habitat (leaf endosphere, root endosphere, and rhizosphere soil; *P* ˂ 0.01). Points are mean dbRDA loadings for each host genotype (*n* = 10) at each sample date (*n* = 8; *n* = 6 for leaf endosphere), and error bars represent ±1 SE of the mean. No data were included for leaf endosphere communities in winter 2018 and 2019 because *Populus* spp. are deciduous trees that do not retain leaves outside the growing season. end., endosphere; rhizo., rhizosphere.

We found that microbial community composition at the amplicon sequence variant (ASV) level differed between seasons and years for bacterial/archaeal communities and fungal communities in each plant-associated habitat (permutational multivariate analysis of variance [PERMANOVA], *P* ˂ 0.001; Fig. 1; Table S1). Furthermore, microbial community composition in a given season depended on year for all communities (season × year interaction, *P* ˂ 0.001; Fig. 1; Table S1). The effects of host species on temporal patterns were idiosyncratic and relatively weak, explaining ˂2% of variation in microbial community composition relative to the cumulative variance explained by season, year, and their interaction (8%–21%; Fig. 1; Table S1). The statistical significance of temporal effects on microbial community composition was identical for repeated measures PERMANOVA (Table S1) and when evaluated using distance-based redundancy analysis (dbRDA) (Fig. 1), demonstrating that our findings were unaffected by the repeated sampling of genetically identical individuals over time or the type of statistical test employed. We also detected a significant effect of season on community dispersion in all communities except bacteria/archaea in the leaf endosphere [analysis of variance (ANOVA), *P* ˂ 0.05; Table S2], suggesting microbial communities may also be more variable among replicates within certain seasons.

**Fig 2:**
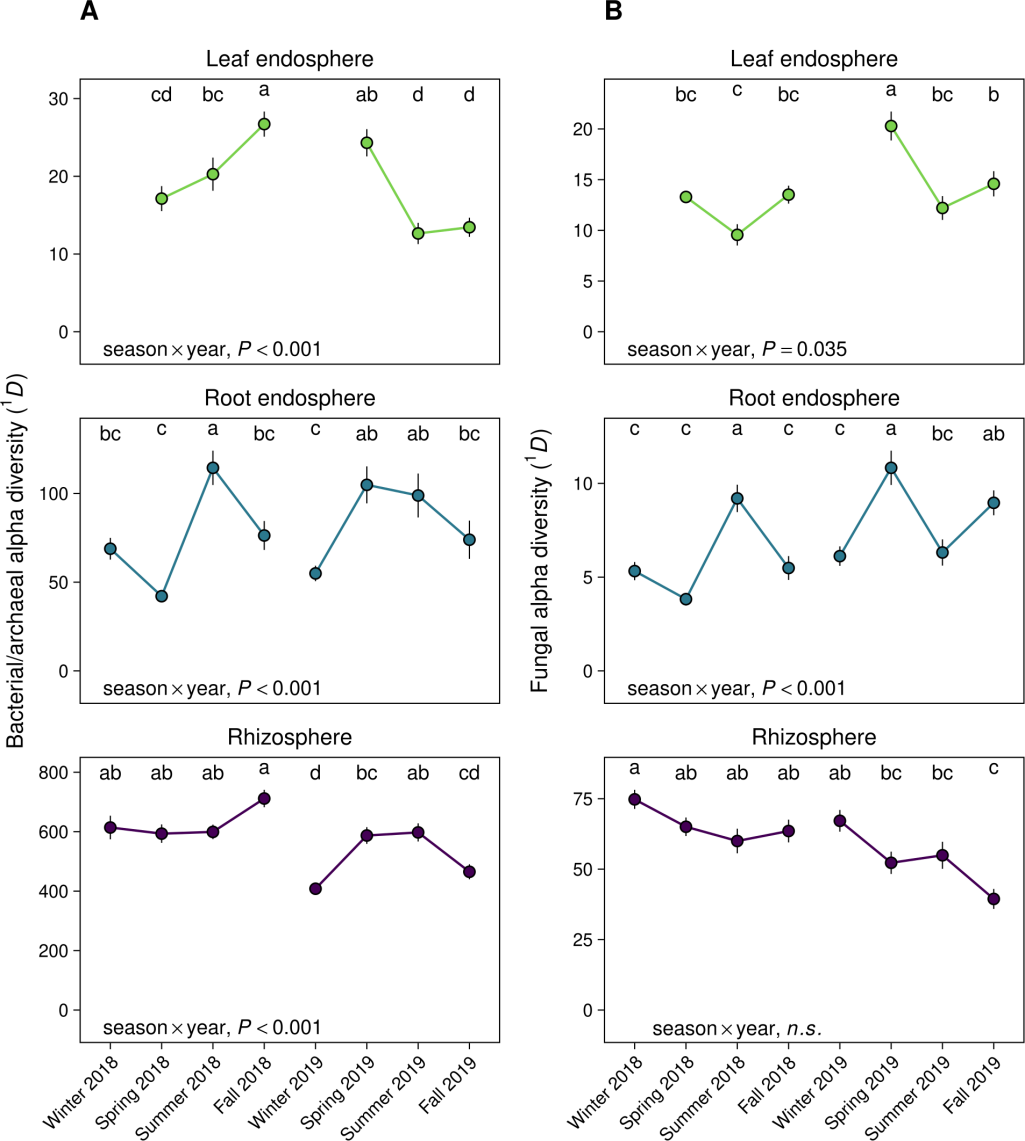
Microbial *α*-diversity (Hill diversity, *q* = 1; ^1^*D*) for bacterial/archaeal communities (**A**) and fungal communities (**B**). *^1^D* accounts for both species richness and the relative abundance of species (i.e., evenness) and is therefore analogous to the Shannon-Weiner diversity index. Points are mean values and error bars represent ±1 SE of the mean. The effect of season, year, host species, and their interactions on ^1^*D* were evaluated using three-way ANOVA (Table S3), and the statistical significance of the interaction between season and year is displayed in each panel. ANOVAs were run for each microbial domain × plant-associated habitat combination. Different lowercase letters represent statistically significant differences between mean values between sample dates within each microbial domain × plant-associated habitat combination, as calculated using Tukey’s honestly significant difference test. No data were included for leaf endosphere communities in winter 2018 and 2019 because *Populus* spp. are deciduous trees that do not retain leaves outside the growing season. n.s., *P* > 0.05.

We calculated microbial *α*-diversity as Hill numbers with *q* = 1 (^1^*D*). The parameter *q* determines the weighting of species relative abundances in diversity calculations. Specifically, *q* = 1 (^1^*D*) accounts for both species richness and the relative abundance of species (i.e., evenness), and it is therefore analogous to the Shannon-Weiner diversity index (46). By contrast, *q* = 0 does not consider species relative abundances and is therefore equivalent to species richness, and *q* = 2 down-weights rare species. We found that seasonal patterns in ^1^*D* were dependent on year (season × year interaction, *P* < 0.05) except for fungal communities in the rhizosphere, where diversity progressively declined across both years (*P* ˂ 0.05; Fig. 2; Table S3). The statistical significance of temporal effects on microbial *α*-diversity was identical for repeated measures ANOVA, except for the significant interaction between season and year for fungal communities in the leaf endosphere (Table S3). Thus, our findings were unaffected by the repeated sampling of genetically identical individuals over time. The effects of host species on temporal patterns in bacterial/archaeal *α*-diversity were weak (*R*^2^ < 0.01) and were absent for fungal communities (*P* > 0.05; Table S3).

### Relationships between microbial community composition and climatic conditions

Climatic conditions differed significantly among seasons (permutational multivariate analysis of variance [PERMANOVA], *R*^2^ = 0.705, *P* ˂ 0.001), but there were no statistically significant effects of year or the interaction between season and year on climatic conditions (*P* > 0.05, Fig. S1B). Climatic conditions were strongly correlated between 2018 and 2019 (Fig. S2A) and exhibited an oscillatory pattern with time between sample dates (Fig. S2B). Thus, variation in climatic conditions primarily corresponded to season, and these seasonal differences were consistent between 2018 and 2019.

To further understand if seasonality was an important component of temporal variation in the *Populus* microbiome, we evaluated whether microbial community composition was related to seasonal variation in climatic conditions using dbRDA (Fig. 1). We found that microbial community composition was significantly related to climatic conditions for bacterial/archaeal and fungal communities in each plant-associated habitat (dbRDA, *P* ˂ 0.01; Fig. 1). Among bacterial/archaeal communities, climatic conditions explained 4.6% of variation in community composition in the leaf endosphere, 2.2% in the root endosphere, and 3.8% in the rhizosphere, whereas climatic variables explained 5.2% of variation in fungal community composition in the leaf endosphere, 2.3% in the root endosphere, and 3.4% in the rhizosphere.

### Long-term changes in the *Populus* microbiome

**Fig 3:**
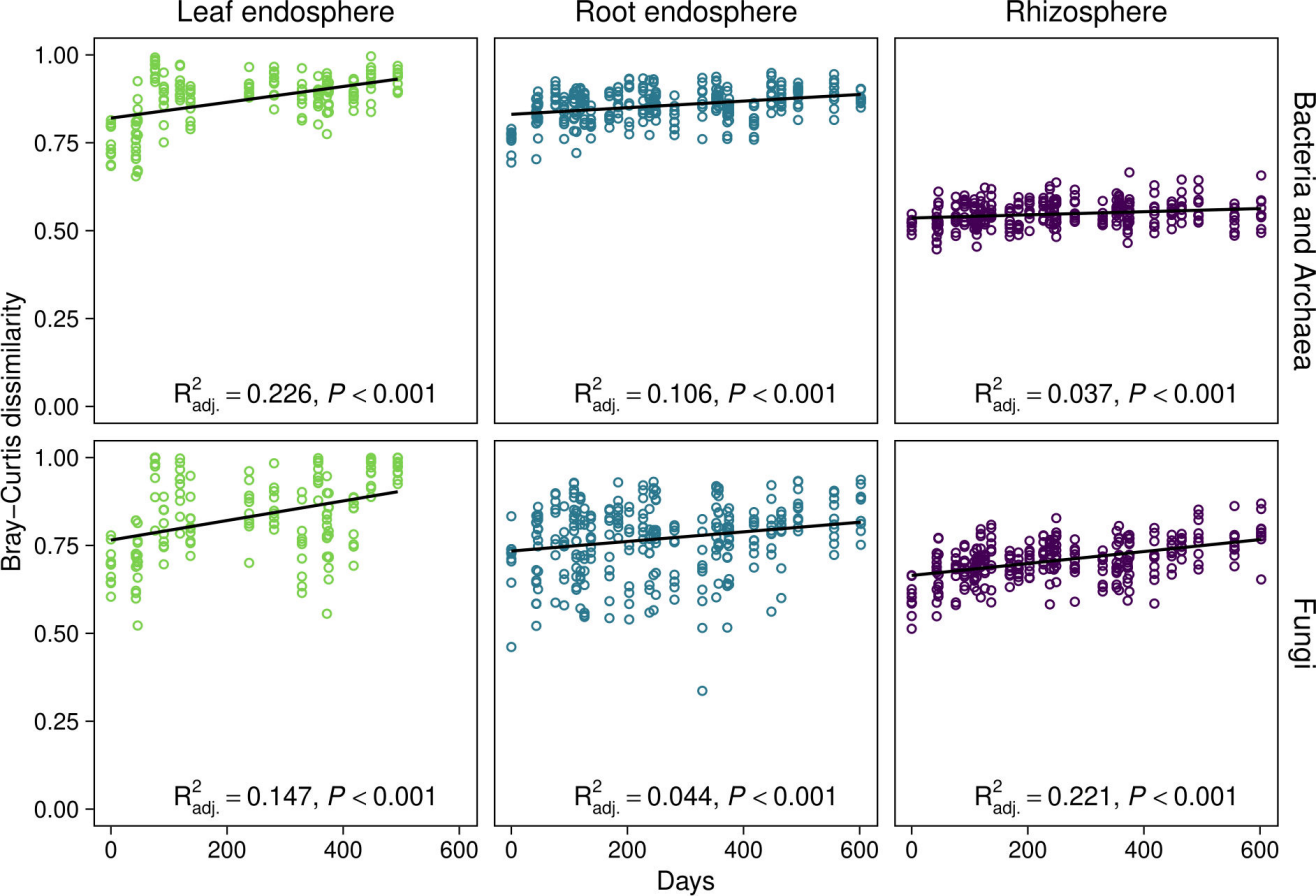
Relationships between Bray-Curtis community dissimilarity and time between sample dates. Each point represents the mean Bray-Curtis dissimilarity for each host genotype between two sampling dates. Trend lines, adjusted *R*^2^ values (*R*^2^_adj_), slopes, and *P* values were obtained from linear regression. Slopes (95% CI) for each bacterial/archaeal community were as follows: leaf endosphere, 0.081 (0.058–0.105); root endosphere, 0.034 (0.023–0.046); and rhizosphere, 0.016 (0.007–0.026). Slopes for each fungal community were as follows: leaf endosphere, 0.101 (0.064–0.138); root endosphere, 0.050 (0.024–0.076); rhizosphere, 0.062 (0.049–0.075). No data were included for leaf endosphere communities in winter 2018 and 2019 because *Populus* spp. are deciduous trees that do not retain leaves outside the growing season.

To understand whether temporal variation in microbial community composition was also driven by longer-term changes related to host age, we used linear regression to evaluate relationships between community dissimilarity and time between sample dates (Fig. 3). Statistically significant positive relationships would demonstrate community dissimilarity progressively increased with time between sampling dates. This directional change in community composition would not be present if seasonal oscillations were the sole driver of community change. Consistent with this possibility, we observed statistically significant positive correlations between community dissimilarity and time between sampling dates for bacterial/archaeal and fungal communities in all plant-associated habitats (linear regression, *P* ˂ 0.001; Fig. 3). These relationships remained statistically significant based on linear mixed effect (LME) models with genotype as a random effect (*P* ˂ 0.001), demonstrating that our findings were unaffected by the repeated sampling of genetically identical individuals over time.

### Microbial groups underpinning temporal patterns

**Fig 4:**
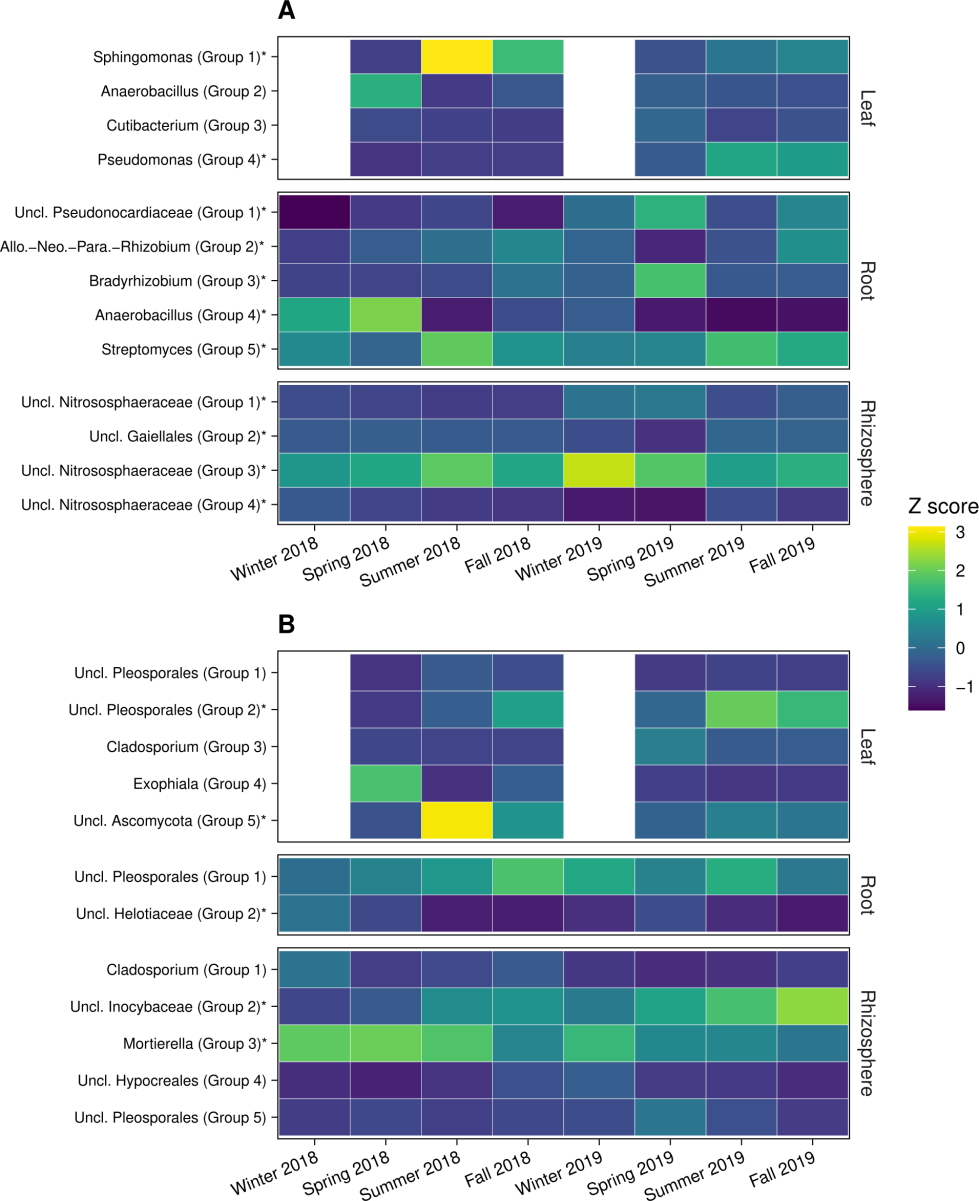
Heatmap of relative abundances for core groups at each sample date (*n* = 8, *n* = 6 for leaf endosphere) for bacterial/archaeal communities (**A**) and fungal communities (**B**). Mean relative abundances were calculated at each time point for each core group across host genotypes (*n* = 10). Mean relative abundances were then centered and scaled as *Z*-scores (mean = 0, SD = 1) within each plant-associated habitat. Group labels include the taxon accounting for the largest percentage of sequences belonging to each core group. No data were included for leaf endosphere communities in winter 2018 and 2019 because *Populus* spp. are deciduous trees that do not retain leaves outside the growing season. Asterisks denotes a significant effect of season on the relative abundance of a core group (ANOVA, *P* ˂ 0.05; Table S5).

To understand which microbial taxa were responsible for the temporal patterns in community composition, we delineated groups of core taxa that exhibited similar temporal dynamics (Fig. 4). Core ASVs accounted for 32%–66% of overall Bray-Curtis dissimilarity. In the leaf endosphere, core bacterial/archaeal groups dominated by *Sphingomonas* (group 1), *Anaerobacillus* (group 2), and *Pseudomonas* (group 4), as well as core fungal groups represented by Pleosporales (group 2), *Exophiala* (group 4), and unclassified Ascomycota (group 5), consistently exhibited the largest shifts in relative abundance between spring and summer of each year (Fig. 4; Tables S4 and S5). By contrast, core bacterial/archaeal groups in the root endosphere oscillated idiosyncratically between seasons, whereas two fungal core groups (primarily Pleosporales [group 1] and Helotiaceae [group 2]) exhibited opposite seasonal patterns each year (Fig. 4; Tables S4 and S5). Nitrososphaeraceae was a prominent member of bacterial/archaeal core groups in the rhizosphere, and the rank order of this group converged by fall of both years (Fig. 4; Tables S4 and S5). Fungal communities in the rhizosphere were characterized by a decline in a group dominated by *Mortierella* (group 3) and an increase in a core group belonging to Inocybaceae (group 2) across years (Fig. 4; Tables S4 and S5). Patterns in the relative abundance of core groups were largely synchronous between *P. deltoides* and *P. trichocarpa* (Pearson’s *r* = 0.850, *P* ˂ 0.001).

**Fig 5:**
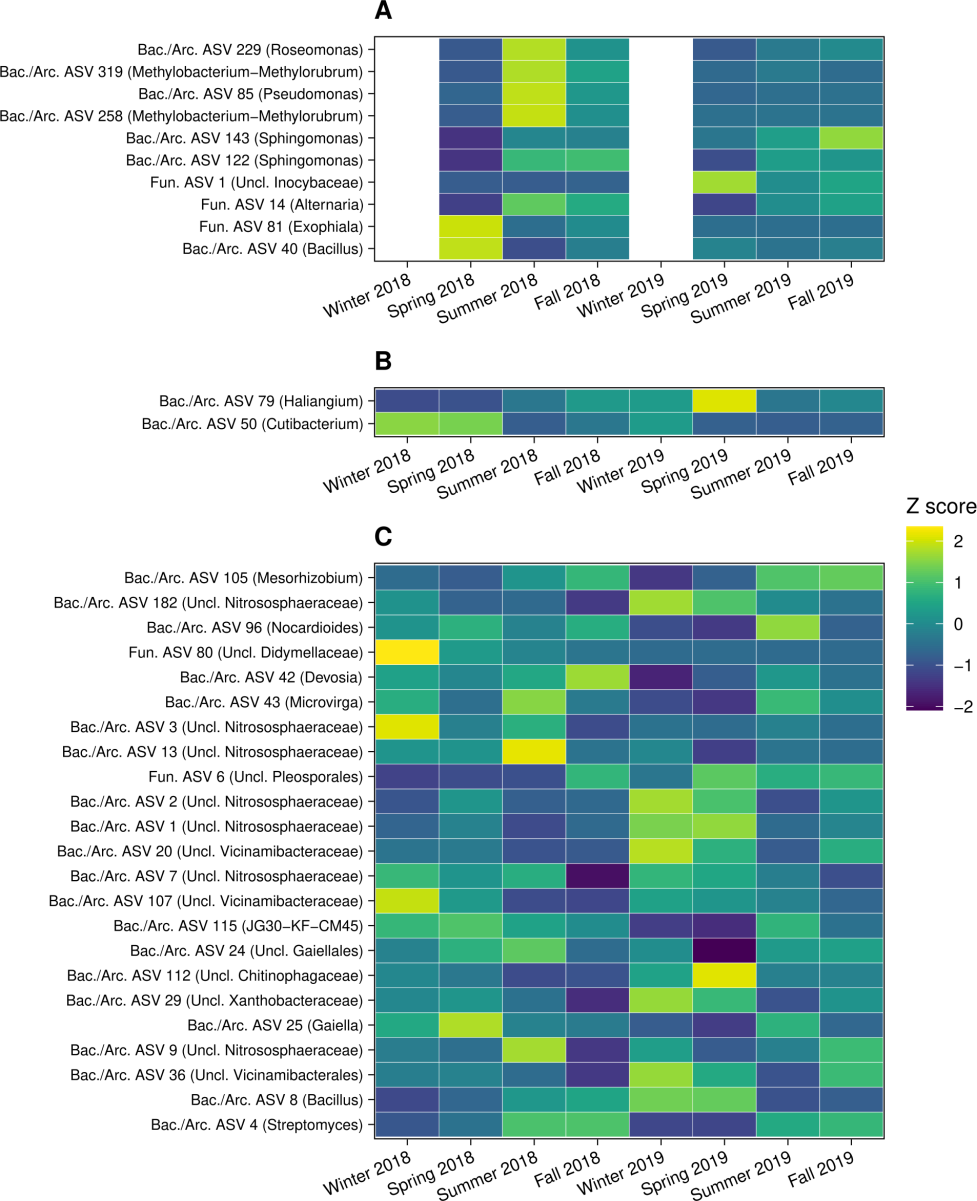
Heatmap of relative abundances for hub ASVs at each sample date (*n* = 8, *n* = 6 for leaf endosphere) for leaf endosphere (**A**), root endosphere (**B**), and rhizosphere communities (**C**). Mean relative abundances were calculated at each time point for each ASV across host genotypes (*n* = 10). Mean relative abundances were then centered and scaled as *Z*-scores (mean = 0, SD = 1) for each ASV. This standardization enables the comparison of temporal patterns between ASVs that may differ substantially in relative abundance. ASV labels include the ASV number and taxonomic assignment at the genus level. No data were included for leaf endosphere communities in winter 2018 and 2019 because *Populus* spp. are deciduous trees that do not retain leaves outside the growing season.

We then used co-occurrence analyses to identify hub taxa that could be central to temporal community turnover through potential species interactions (see Table S6 for network characteristics). The five most connected hub ASVs in the leaf endosphere belonged to *Methylobacterium*-*Methylorubrum* (two ASVs), *Sphingomonas*, *Bacillus*, and *Pseudomonas*, whereas the most connected hub ASV in the root endosphere was classified as *Cutibacterium* (Fig. 5; Table S7). Seven of 23 hub ASVs in the rhizosphere belonged to the archaeal family Nitrososphaeraceae (Fig. 5; Table S7). Seasonal patterns in the mean relative abundance of 89% of hub ASVs differed between years (Fig. 5), and these patterns were consistent between *P. deltoides and P. trichocarpa* (Pearson’s *r* = 0.66, *P* < 0.001).

**Fig 6:**
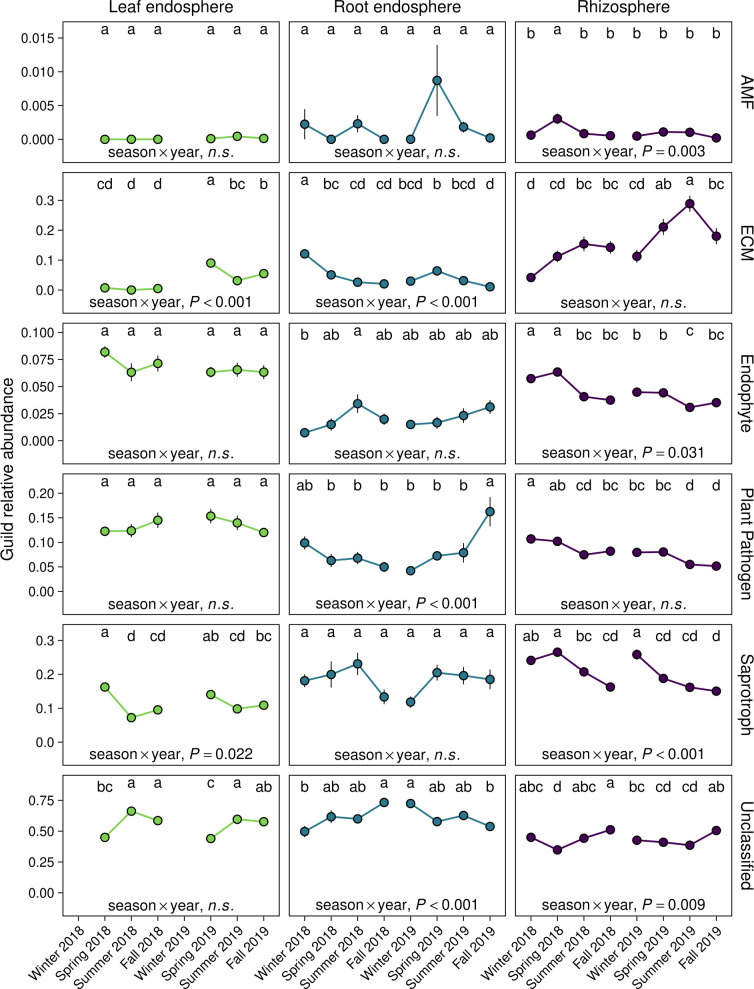
Relative abundance of fungal guilds at each sample date (*n* = 8, *n* = 6 for leaf endosphere). Points are mean values and error bars represent ± 1 SE of the mean. The effect of season, year, and host species, and their interactions on the relative abundance of each guild were evaluated using three-way ANOVA. The statistical significance of the interaction between season and year is displayed in each panel. ANOVAs were run on each guild for each microbial domain × plant-associated habitat combination. Different lowercase letters represent statistically significant differences between mean values between sample dates within each microbial domain × plant-associated habitat combination, as calculated using Tukey’s honestly significant difference test. n.s., *P* > 0.05. No data were included for leaf endosphere communities in winter 2018 and 2019 because *Populus* spp. are deciduous trees that do not retain leaves outside the growing season. AMF, arbuscular mycorrhizal fungi; ECM, ectomycorrhizal fungi.

Finally, we assessed how fungal functional groups differed over time to obtain insights into the functional consequences of temporal shifts in fungal community composition. The relative abundances of most fungal guilds were constant over time in the leaf endosphere and root endosphere (Fig. 6; Table S8). However, we observed an increase in the abundance of functionally unclassified fungi in the leaf endosphere from spring to summer of both 2018 and 2019, as well as an increase in the relative abundance of plant pathogens in fall 2019 in the root endosphere (*P* ˂ 0.05; Fig. 6; Table S8). Rhizosphere fungal communities exhibited a long-term increase in the relative abundance of ectomycorrhizal fungi and a decline in plant pathogens across the two years, while the relative abundance of saprotrophic fungi declined throughout the year in both 2018 and 2019 (Fig. 6; Table S8). These responses likely explain the strong directional change in fungal composition (Fig. 1F and 3) and the decline in fungal diversity (Fig. 2B) we observed in the rhizosphere. Patterns in fungal guilds were largely consistent across host species (season × year × host species, *P* ˃ 0.05; Table S8).

## DISCUSSION

The central role of microorganisms in plant health (17, 47–49) has motivated decades of research on the assembly of plant microbiomes (50). It is now clear that plant microbiomes comprise a subset of microbial communities from the immediate surrounding environment (38, 51), and previous work has advocated using unified community ecology frameworks (e.g., see references 52, 53) to understand the ecological processes through which plant-associated subsets assemble from broader microbial species pools (40, 54). Empirical tests of these frameworks suggest a complex mixture of ecological selection, drift, and dispersal structure plant microbiomes, and the climatic, host genetic, and edaphic drivers of selection vary considerably in their identity and relative importance among plant species and ecosystems (17, 40, 55). This context dependency has slowed the establishment of general principles to describe the assembly of the plant microbiome (50), which limits our ability to predict how microbiomes and their host plants will respond to environmental change (16) and manipulate the plant microbiome to improve agricultural production and sustainability (56, 57). Our study begins to address this knowledge gap by showing seasonal dynamics and longer-term changes simultaneously shape the temporal assembly of tree microbiomes, generating unique seasonal patterns each year and resulting in progressive microbial community change as host trees age (Fig. 1 to 3).

Microbial community composition exhibited clear seasonal patterns in the leaf endosphere, root endosphere, and rhizosphere of *Populus* (Fig. 1 to 3). In addition to significantly differing among seasons, temporal variation in microbial community composition was correlated with seasonal shifts in climatic conditions (Fig. 1). Climate shapes spatial patterns in plant microbiomes (17, 58, 59), and our observations suggest seasonal climatic variation also influences temporal patterns in the plant microbiome. We found that climatic conditions explained more variation in microbial community composition for communities in the leaf endosphere than in the root endosphere and rhizosphere (Fig. 1), which aligns with evidence that aboveground and belowground plant responses to the environment often differ (60). Furthermore, our observation that microbial communities inhabiting the leaf endosphere may be more sensitive to seasonal variation in climatic conditions than fine root and rhizosphere communities (Fig. 1) could have important implications for our understanding of how plant microbiomes respond to ongoing environmental change (16, 41). Season alone explained relatively low proportions of variation in microbial community composition (˂10%, Table S1), suggesting other factors also shape temporal patterns in the *Populus* microbiome. Nonetheless, our results emphasize that, like grasses and herbaceous plants (21, 23, 24), seasonal variation is an important component of temporal dynamics in the microbiomes of trees.

We also found that the composition of the *Populus* microbiome in a given season depended on year (Fig. 1), and our results suggest longer-term changes in microbial community composition may modify the seasonal assembly of microbial communities. The composition of the *Populus* microbiome shifts considerably throughout the first year of growth (36), and these changes may continue as trees undergo metabolic shifts throughout development (31, 32, 61). Consistent with this possibility, we found microbial community composition differed between years (Fig. 1) and became progressively dissimilar with increasing time between sample dates (Fig. 3), indicating microbial community composition continued to change directionally across the second and third years of growth. Furthermore, climatic conditions were highly consistent between years (Fig. S1 and S2) and exhibited a cyclical (i.e., seasonal) relationship with time between sample dates (Fig. S2B). This pattern suggests the overall increase of community dissimilarity with greater time between sampling dates (Fig. 3) was driven by longer-term changes corresponding to host age or other unmeasured abiotic and biotic factors rather than seasonal variation in climatic conditions. Consequently, the difference in seasonal patterns between years (significant season × year interactions; Fig. 1; Table S1) likely arose because microbial communities were also shifting over longer time spans. Seasonality and compositional shifts that track host development or are driven by microbial succession (34) are important aspects of temporal variation in plant microbiomes, but these dynamics have generally been considered separately (23, 32). Our findings provide novel insights into how temporal patterns in plant microbiomes emerge from the combined effects of seasonality and longer-term changes, which together explained considerable variation in microbial community composition (up to 21%, Table S1).

The annual re-assembly of leaf endosphere communities could also explain interannual dissimilarity in microbial community composition (Fig. 1A and B). Specifically, the leaves of deciduous trees are produced and senesce synchronously on an annual basis, whereas fine roots turn over throughout the growing season (62, 63), and the soil in which the rhizosphere forms remains physically stable. Microorganisms colonize newly formed plant tissues through vertical transmission (64) and dispersal from adjacent tissues and the environment (e.g., air and soil; see references 17, 40), and stochasticity in the order of early species arrival to plant habitats can generate priority effects that lead to diverging microbial community outcomes (54, 65, 66). Consequently, priority effects could generate interannual differences in the trajectory of microbial community assembly in the leaf endosphere, such as those we observed in leaf endosphere communities in our study (Fig. 1A and B). However, directly testing the roles of priority effects in the *Populus* microbiome will require the experimental manipulation of microbial species arrival (67–69).

Many core and hub taxa that drove temporal dynamics belonged to culturable microbial lineages associated with *Populus* (Fig. 4 and 5; Tables S4 and S7). For example, isolates belonging to *Pseudomonas* frequently dominate culture-based studies of *Populus* microbiomes (70, 71), and this genus was particularly abundant among core and hub ASVs in the leaf endosphere (Table S7). *Streptomyces* and *Rhizobium* were key core genera in the root endosphere, and *Bacillus* accounted for core ASVs in all plant-associated habitats (Tables S4 and S7). Because the function of bacterial taxa within the same genus can depend on plant-associated habitat (e.g., see reference 72), understanding function will require experimental investigation of these lineages in a specific habitat. *Pseudomonas*, *Streptomyces*, *Rhizobium*, and *Bacillus* are represented in reproducible synthetic communities for *Populus* (70, 73), which will facilitate experimental efforts to validate the temporal dynamics we observed and their potential impacts on host health. Among fungi, several core and hub taxa belonged to Pleosporales, a taxonomically diverse order of saprotrophic, endophytic, and pathogenic species (74). Many Pleosporales we detected were not classified beyond the order level (Tables S4 and S7), and it will therefore be important to leverage the growing number of Pleosporales isolates from *Populus* (75) to understand the functional consequences of these taxa.

Temporal patterns in rhizosphere communities were driven by microbial groups that could be involved in plant nutrient acquisition. For example, the rank order of core groups dominated by Nitrososphaeraceae (Fig. 4; Table S4), which are ammonia-oxidizing archaea that mediate the rate-limiting step of nitrification (76, 77), converged by the fall of both years despite differing in composition at the beginning of each year (*P* ˂ 0.05; Fig. 4; Table S5). Some *Populus* spp. preferentially acquire nitrate over ammonium (78), and annual convergence to a similar composition of ammonia-oxidizing archaea (Fig. 4) could therefore influence plant nitrogen nutrition. Additionally, nitrite-oxidizing bacteria in the order Nitrospirales (79) differed in response to season, year, and their interaction (ANOVA, *P* ˂ 0.001) and were positively correlated with groups 1 and 3 and negatively correlated with group 4 (Pearson’s correlation, *P* ˂ 0.001). These relationships between ammonia-oxidizing archaea and nitrite-oxidizing bacteria suggest temporal dynamics in rhizosphere communities could have important implications for nitrification. We also found that the relative abundance of ectomycorrhizal fungi—particularly those belonging to the family Inocybaceae (80)—peaked during the summer and exhibited a long-term increase across 2018 and 2019 (Fig. 4 and 6). The relative abundance of ectomycorrhizal fungi typically increases during seasons with high photosynthetic activity (20, 35, 81), likely explaining why relative abundance of these fungi was greatest during summer (Fig. 6). The capacity of trees to supply ectomycorrhizal fungi with photosynthate also tends to increase as trees become larger through mid-age (82), which could be responsible for the longer-term increase in ectomycorrhizal fungi we observed in the rhizosphere (Fig. 4 and 6). Furthermore, this ectomycorrhizal response occurred with a concomitant decline in fungal plant pathogens and the fungal genus *Mortierella* (Fig. 4 and 6), which includes many root-associated species (75) and saprotrophs that are common in decaying fine root litter and soil (83, 84). Thus, the directional change in fungal communities we observed (Fig. 1 to 3) likely reflects a shift from fungal pathogens and saprotrophs toward greater potential for nutrient acquisition by ectomycorrhizal fungi. Ectomycorrhizal fungi also influence soil organic matter formation and decomposition (85–87), suggesting temporal variation in the relative abundance of these plant symbionts could have important consequences for seasonal and long-term soil carbon dynamics.

Our findings must be interpreted in light of four important considerations. First, we found weak effects of host species on seasonal variation in the *Populus* microbiome. This observation aligns with other studies finding weak genotypic effects on the composition of plant microbiomes (88) but is somewhat surprising, given previous evidence for differences in microbial composition among *Populus* genotypes (including at the same site) (36, 38) and in greenhouse studies (39, 89). It is possible that our unbalanced study design (eight genotypes of *P. trichocarpa* and two genotypes of *P. deltoides*) did not provide sufficient power to fully separate the effects of host species on seasonal variation in microbial communities. Nonetheless, seasonal patterns were similar between these two geographically widespread species of *Populus*, lending generality to our findings. Second, relic DNA can obscure temporal patterns in microbial community composition (90). However, the effect of relic DNA on patterns in microbial community composition remains in question (91). Moreover, microbial composition was highly dynamic across sample dates (Fig. 1 to 6), suggesting we captured a considerable component of temporal variation in the *Populus* microbiome. Third, we cannot separate whether longer-term changes in the *Populus* microbiome track host development (32), occur due to successional processes within microbial communities (34), or both. Finally, our inference about longer-term development of the *Populus* microbiome is based on 2 years of observations early in host development, whereas individuals of this genus can live over 100 years (92). Space-for-time substitutions have revealed temporal turnover in tree microbiomes with increasing stand age (32, 93), and the clonal reproduction of many *Populus* spp. in natural ecosystems (94) can provide a unique opportunity to further refine our understanding of these dynamics by concurrently observing them across the “lifetime” of genetically identical individuals.

By experimentally isolating temporal variation in the *Populus* microbiome, our study demonstrated that seasonality and longer-term shifts in microbial community composition interact to control the temporal assembly of microbial communities associated with an ecologically and economically important tree genus. However, 74%–89% of variation in microbial community composition within each plant-associated habitat remained unexplained by temporal variables and host species (Table S1). Species interactions, such as competition, could play an important role in temporal patterns in ecological communities (4, 5), and our identification of several hub taxa suggests microbial interactions could drive temporal patterns in the *Populus* microbiome (Fig. 5). Verification of these putative species interactions (95) may be an effective way to reduce unexplained variation in microbial community composition. Additionally, the functional consequences of seasonal variation in plant microbiomes remain an important knowledge gap (23), and we identified temporally dynamic taxa in the *Populus* rhizosphere that could have important implications for nutrient cycling. Metagenomic analyses have recently expanded our understanding of archaeal ammonia oxidation (77, 96) and nutrient acquisition by ectomycorrhizal fungi (97), and these approaches could provide novel insights into the functional consequences of temporal variation in ammonia-oxidizing archaea and ectomycorrhizal fungi in the *Populus* rhizosphere. Finally, establishing background temporal variation is crucial for understanding current and future patterns in community composition (98), and our findings indicate that seasonality and longer-term changes must be accounted for when attempting to understand spatial controls over the assembly of tree microbiomes and their temporal responses to ongoing environmental change.

## MATERIALS AND METHODS

### Collection of plant cuttings

We used a common garden approach to isolate temporal changes in microbial community composition while eliminating spatial variation in environmental conditions. We also used the ability to clonally propagate *Populus* to control for host genetic effects. To obtain plants for the common garden experiment, dormant cuttings of eight *Populus trichocarpa* genotypes were collected from the Corvallis, OR, and Clatskanie, OR, genome-wide association study populations (99) in January 2017, and two *P. deltoides* genotypes were collected from a previous study at the site of this experiment in Blount County, TN (36, 38, 100). Briefly, this site was located at N 35°50′39″ and W 83°57′36″, on soil classified as inceptisols of the Emory series with a silt loam A horizon from 0 to 25 cm and a silty clay loam B horizon beginning at 25 cm. Cuttings were shipped overnight on ice and maintained at 4°C until propagation in March 2017. We surface sterilized cuttings in a 1% Zerotol 2.0 solution and placed rooting powder (0.1% indole-3-butyric acid) on sterile cutting surfaces. We placed the plants in autoclaved potting soil (Fafard 52 Mix; Sun Gro Horticulture, Massachusetts, USA) to minimize the transfer of microbial communities from the greenhouse, allowed them to establish for ~2 months, and transferred them to an experimental plot in 2017 in Blount County, TN (38), after leaf buds opened and sufficient root growth occurred.

### Experimental design and sample collection

We planted 80 *Populus* individuals (10 genotypes × 8 individuals per genotype) using a soil auger (10-cm diameter holes to a depth of 30 cm) to minimize the disruption of soil in the rooting zone. Each plant was separated by 1 m, and genotypes and species were approximately evenly divided among three blocks. We collected samples in winter (19 February 2018 and 7 February 2019), spring (7 June 2018 and 30 May 2019), summer (22 August 2018 and 29 August 2019), and fall (4 October 2018 and 14 October 2019) of 2018 and 2019, and we ensured sample dates for each season were ~1 year apart (±12 days). At each sample date, we randomly selected three to five replicate plants (at least one from each block) for each genotype. We collected ~3 leaves per plant, obtained ~3 g of fine roots (≤2 mm diameter) per time point by gently excavating roots and attached soil from the upper 10 cm of soil using a hand trowel, and ensuring fine roots were traced back to the target tree prior to collection. No leaves were collected during the winter season because *Populus* spp. are deciduous and do not retain leaves outside the growing season. We transported leaf samples to Oak Ridge National Laboratory on ice before processing and transferred roots and attached rhizosphere soils on dry ice to be stored at −80°C prior to processing as described previously (38). Briefly, we surface sterilized leaves with 3% bleach followed by four rinses with sterile deionized water to obtain a “leaf endosphere” sample. We composited the three leaves per individual per time point into a single sample and homogenized the tissue prior to DNA extraction. We rinsed fine roots of adhering soil with sterile deionized water and centrifuged the suspension to collect rhizosphere soil. The rinsed fine roots were then surface sterilized using a 100% ethanol rinse followed by 3% bleach, and four rinses with sterile deionized water. This surface sterilization ensured we obtained a “root endosphere” sample. Fine roots (≤2 mm diameter) were cut into small (˂5 mm long) segments and homogenized prior to DNA extractions. We plated 50 µL of the final rinsate for both leaves and fine roots on LB and R2A media and incubated plates at room temperature for 2 days to ensure surface sterility of target tissues, and repeated the sterilization procedure with a fresh sample if colonies were observed (38). It is important to note that this method does not confirm that all free DNA, dead cells, or unculturable microbes were removed from the surface of plant tissue.

### Environmental conditions

To characterize seasonal variation in climatic conditions, we obtained daily surface weather conditions from the DAYMET daily surface weather and climatological summaries database (1-km^2^ resolution; see reference 101). We included six climatic variables (minimum temperature, maximum temperature, day length, short wave radiation, precipitation, and vapor pressure; Fig. S1A), using the bounding box coordinates for the location of our common garden experiment (W 83.9566°, S 35.8396°, E 83.9479°, N 35.8438°). Because there was strong covariation among several variables (Fig. S1 and S2A), we used principal components analysis (PCA) loadings calculated from the six climate variables as a metric of seasonal differences in environmental conditions. To calculate these loadings, we used the 7 days prior to each sample date as the values to represent each season after scaling each variable between 0 and 1 to account for different units.

### DNA isolation, amplicon sequencing, and sequence processing

Frozen plant material was ground into a fine powder on liquid nitrogen, and we extracted DNA from 50-mg fine roots and leaf tissue per sample using the Mag-Bind Plant DNA Plus 96 Kit (Omega Bio-Tek) and 250-mg rhizosphere soil using the Mag-Bind Environmental DNA 96 Kit (Omega Bio-Tek). The quality and quantity of extracted DNA were assessed using a Nanodrop 1000 spectrophotometer (NanoDrop Products, Wilmington, DE, USA) and Qubit Fluorometer with the dsDNA BR assay kit (Invitrogen, Waltham, MA, USA).

We used a two-step PCR approach with barcode-tagged primers and templates to amplify the V4 region of the 16S rRNA gene for bacteria and archaea, as well as the ITS2 region of the internal transcribed spacer for fungi, using pooled primer sets to increase the taxonomic coverage of archaeal, bacterial, and fungal communities (38, 102). To reduce the amplification of plant DNA, we included 2.5-µM peptide nucleic acid blockers in the first PCR step (GGCAAGTCTTCTTCGGA and GGCTCAACCCTGGACAG for 16S PCR and CGAGGGCACGTCTGCCTG for ITS2 PCR). Each PCR reaction consisted of 0.25 µM of the primer pair, 1× KAPA HiFi HotStart ReadyMix (Roche Molecular Systems), 2-µL template DNA, and brought to a total volume of 25 *µ*L with molecular-grade water. Reaction conditions for the first PCR step consisted of an initial denaturation at 95°C for 3 min, followed by 25 cycles of 95°C for 30 s, 78°C for 30 s, 55°C for 30 s and 72°C for 30 s, with a final extension of 72°C for 5 min. We amplified endosphere samples for 30 cycles to obtain sufficient yields. We performed the second PCR step according to the Illumina 16S sequencing library preparation instructions using an initial denaturation step of 95°C for 3 min, followed by eight cycles of 95°C for 30 s, 55°C for 30 s, and 72°C for 30 s, and a final extension of 72°C for 5 min. Following PCR, we purified and then pooled PCR products (Agencourt AMPure XP beads; 0.7:1 bead-to-DNA ratio; Beckman Coulter, Inc., Pasadena, CA, USA), and sequenced them on an Illumina MiSeq instrument (2 × 251 cycles). We included a 15% PhiX spike due to low sequence diversity.

We removed primers and adaptors using Cutadapt (103) and extracted the ITS region from fungal reads using ITSxpress (104). We delineated bacterial/archaeal and fungal sequences into amplicon sequence variants using DADA2 (105), removing forward and reverse reads that contained ˃2 expected errors based on error estimation that accounts for read quality and run-specific sequence error rates. This step also removed merged reads that did not overlap by at least 12 bp or were chimeric. All sequence processing steps were implemented in QIIME2 (version 2022.8) (106). We assigned taxonomic classifications using the naive Bayes classifier and sklearn Python package for 16S sequences against the SILVA database (release 138; see reference 107) with a confidence of 0.7. We assigned taxonomic classifications to ITS2 sequences using consensus BLAST (identity, 80%; *E* value, 0.001; minimum fraction of assignments, 0.51) and the UNITE reference database (version 8.0; see references 108, 109).

Our 16S sequencing effort generated 83,797,412 raw reads, 74% of which (62,387,197) were retained as high-quality joined reads. Of the high-quality reads, 47,269,875 (76%) were assigned to bacteria/archaea after removal of chloroplast and mitochondrial sequences [median (min, max): 7,664 (502, 237,744) in the leaf endosphere, 16,699 (2,429, 354,877) in the root endosphere, and 59,530 [7,652, 345,838] in the rhizosphere]. We detected 46,562 bacterial/archaeal ASVs (2,512 in the leaf endosphere, 17,636 in the root endosphere, and 35,937 in the rhizosphere). Our ITS sequencing effort generated 75,496,559 raw reads, 45% of which (33,986,466) were retained as high-quality joined reads. Of the high-quality reads, 31,805,862 (94%) were assigned to fungi [median (min–max): 13,937 (920–70,554) in the leaf endosphere, 13,804 (550–116,964) in the root endosphere, and 40,734 (5,508–813,423) in the rhizosphere]. We detected 11,497 fungal ASVs (2,410 in the leaf endosphere, 1,591 in the root endosphere, and 9,431 in the rhizosphere).

We used subsampling with ranked scaling (110) to subsample ASV tables to the sequence count of the smallest sample. We subsampled ASV tables to 502 (bacteria/archaea) and 920 (fungi) sequences for the leaf endosphere, 2,429 (bacteria/archaea) and 550 (fungi) sequences for the root endosphere, and 7,652 (bacteria/archaea) and 5,505 (fungi) sequences for the rhizosphere. Microbial communities in the *Populus* endosphere are characterized by far lower microbial biomass (and, thus, lower sequence yields) and diversity than rhizosphere communities (38), and it is therefore appropriate to subsample these communities to different sequence depths to accurately capture differences in microbial community composition and diversity (e.g., see references 36, 102). We emphasize that our study focused on temporal patterns within plant-associated habitats rather than comparisons of microbial community composition and diversity between plant-associated habitats. Thus, comparisons were exclusively made between communities that were subsampled to identical sequence depths. We assigned fungal ASVs to functional guilds using FUNGuild (111).

### Core communities and cross-domain co-occurrence networks

We identified core ASVs to understand which microbial taxa were responsible for temporal patterns in the *Populus* microbiome. Specifically, we selected core ASVs based on abundance-occupancy distributions (15) calculated for each community (i.e., bacteria/archaea and fungi) within each habitat. We retained ASVs present in ≥70% of samples in a habitat in at least one time point to focus our analyses on core ASVs that drove temporal variation in community composition rather than variation among genotypes (21). We determined the contribution (*C*) of these core ASVs to within- and among-season Bray-Curtis dissimilarity (BC) in community composition (112):


$$C=\frac{BC_{core}}{BC_{all}}$$

We delineated groups of core ASVs that varied similarly over time by clustering relative abundance patterns. Specifically, we scaled and centered the relative abundance of each core ASV to a mean of 0 and a standard deviation of 1 (*Z*-scores) and subsequently calculated a distance matrix based on correlations between standardized ASV relative abundances [distance metric of 1 – Pearson’s *r*; see references 21, 113). We grouped ASVs from these distance matrices using agglomerative hierarchical clustering with the complete linkage method implemented with the “hclust” function in R, and selected the minimum number of groups at which variation explained no longer increased appreciably with additional groups (21, 113).

Additionally, we used co-occurrence analyses to detect hub (i.e., highly connected) ASVs that may be important for shaping temporal variation in the *Populus* microbiome through putative species interactions (25, 114). First, we separately filtered six non-subsampled ASV tables (bacteria/archaea and fungi in leaf endosphere, root endosphere, and rhizosphere) by removing ASVs present in ˂20% of samples to reduce the detection of spurious associations. We then used the SPIEC-EASI package, which requires non-subsampled ASV tables, to combine bacterial/archaeal and fungal communities, and estimated co-occurrence networks that were robust to the compositional and sparse nature of microbial community data (115, 116). This produced one cross-domain network for each plant-associated habitat. We classified hub ASVs as those ≥90th percentile for both degree and betweenness centrality (25, 114), and filtered them to include hub ASVs that also belonged to core communities and accounted for ≥0.1% of sequences in their respective plant-associated habitat.

### Statistical analyses

We used PERMANOVA to test for differences in microbial community composition (117) using the “adonis2” function in the “vegan” package (118). PERMANOVA tests were performed for each community (bacteria/archaea and fungi) and plant-associated habitat using Bray-Curtis dissimilarity calculated from subsampled ASV tables as the response variable, and season, year, host species, and their interaction as predictor variables. To ensure that the repeated temporal sampling of genetically identical individuals did not bias our results, we also performed PERMANOVA tests using a repeated-measures approach by constraining permutations within each genotype. We tested the homogeneity of multivariate dispersion with PERMDISP (119) in vegan (“betadisper” function).

We visualized temporal shifts in community composition using constrained ordinations generated with dbRDA. We constructed dbRDA ordinations from Bray-Curtis dissimilarity of ASV-level microbial community composition, and we used season, year, host species, and two environmental PCA axes (PC1 and PC2) as constraining variables in these analyses. We used the statistical tests for constraining variables in dbRDA to evaluate the statistical significance of relationships between microbial community compositions and seasonal variation in environmental conditions. We used PCA (Fig. S1B) to determine composite environmental variables due to strong covariance between several individual environmental variables (Fig. S2A).

We calculated microbial *α*-diversity using Hill numbers (46) with ASVs weighted according to relative abundance (*q* = 1), which differs from diversity calculated from equally weighted ASVs (i.e., species richness; *q* = 0) and down-weighted rare ASVs (*q* = 2). To test for differences in seasonal patterns in microbial *α*-diversity, core group relative abundances, and fungal functional groups, we used three-way ANOVA with season, year, host species, and their interactions as fixed effects. Between group comparisons were tested with Tukey’s honestly significant difference test. We ensured that the repeated temporal sampling of genetically identical individuals did not bias our results by performing repeated-measures ANOVA constrained by genotype.

To evaluate longer-term relationships between microbial community composition and time, we regressed the Bray-Curtis community dissimilarity against time between sample dates (*sensu* 120, 121). We tested the statistical significance of these relationships using linear regression to understand whether microbial community composition exhibited directional changes over time (i.e., positive relationship between dissimilarity and time). To ensure that the repeated temporal sampling of genetically identical individuals did not bias the relationships we detected between microbial community dissimilarity and time between sampling dates, we also performed LME models with genotype as a random effect. Additionally, we used generalized additive models (GAM) to fit a relationship between environmental dissimilarity and time between sample dates to compare this relationship to that of microbial community dissimilarity. We accepted statistical significance at α = 0.05, and all statistical analyses were performed using R (version 4.3.1) (122) in RStudio (version 2023.06.0+421) (123).

## Data Availability

Sequence data are available in the Sequence Read Archive (BioProject accession no. PRJNA993999), and all other data and code are available on Github (https://github.com/argiroff/populus_seasonal).

## SUPPLEMENTAL MATERIAL

Supporting information msystems.00886-23-s0001.docx Supplemental tables and figures.

ASM does not own the copyrights to Supplemental Material that may be linked to, or accessed through, an article. The authors have granted ASM a non-exclusive, world-wide license to publish the Supplemental Material files. Please contact the corresponding author directly for reuse.
