# Supplementary material for: Seasonality and longer-term development generate temporal dynamics in the Populus microbiome
Source: mSystems. 2024 Feb 29;9(3):e00886-23. doi: 10.1128/msystems.00886-23 (PMC10949431; doi:10.1128/msystems.00886-23)
Supplement: Supporting information — Supplemental tables and figures. [file msystems.00886-23-s0001.docx]

**Supporting Information for:** Seasonality and longer-term development generate temporal dynamics in the *Populus* microbiome

William A. Argiroff, Alyssa A. Carrell, Dawn M. Klingeman, Nicholas C. Dove, Wellington Muchero, Allison M. Veach, Toni Wahl, Steven J. Lebreux, Amber B. Webb, Kellie Peyton, Christopher W. Schadt, and Melissa A. Cregger

This document includes:

- Figures S1-S2 (pp. 2-4)
- Tables S1-S8 (pp. 5-23)


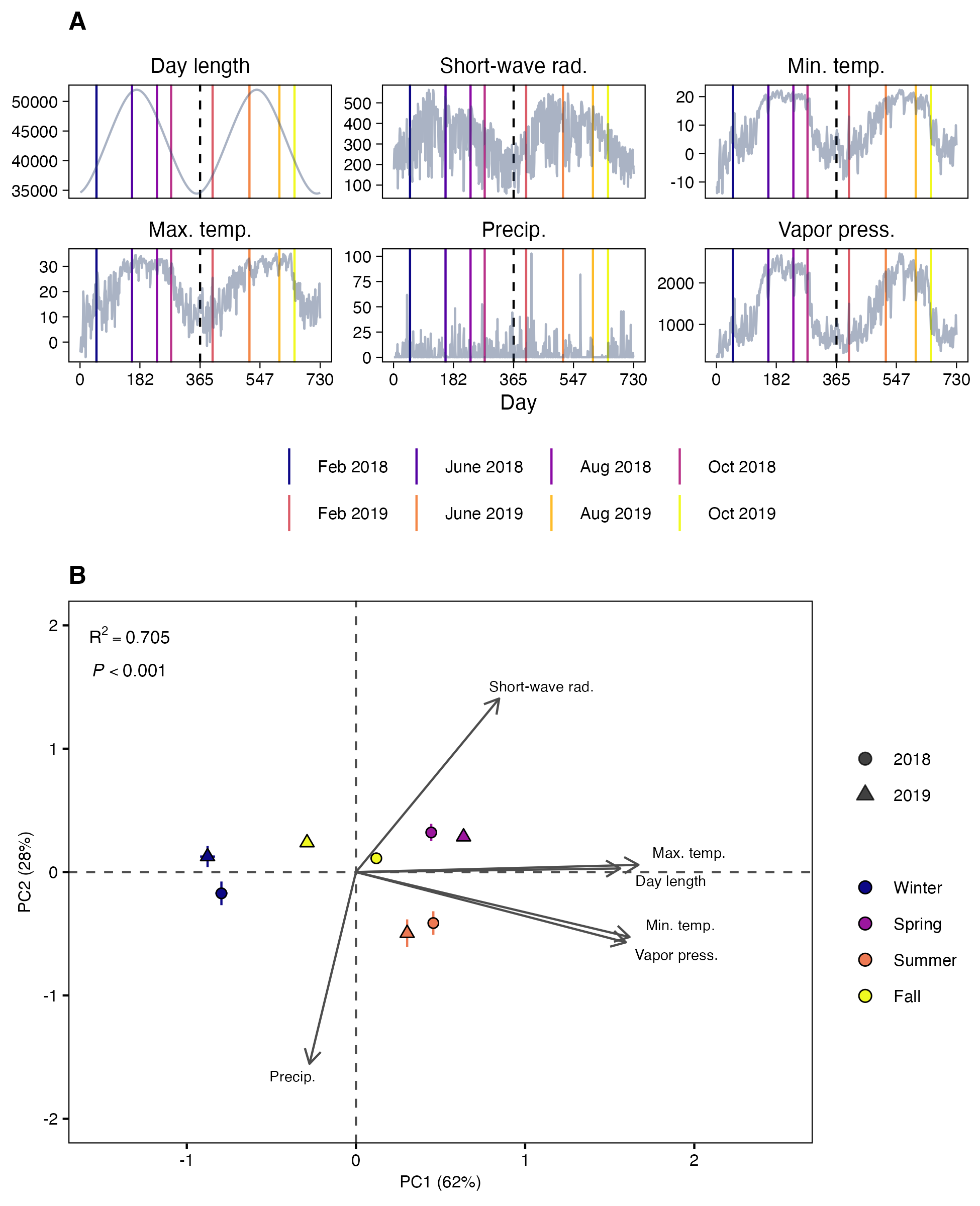


**Fig. S1:** Time series of climatic conditions in 2018 and 2019 (**A**) and ordination based on principal components analysis (PCA) of climatic conditions by season (**B**). Time series were constructed from weekly means. PCA was performed on a Euclidean distance matrix of climatic conditions for the 7 days preceding each sampling date after scaling each variable from 0 to 1. Points represent mean (*n* $=$ 14) of PCA loadings for each sampling date (*n* $=$ 8), and error bars represent one S.E. of the mean. R^2^ and *P* values were determined using PERMANOVA and represent the variation in climatic conditions explained by season.


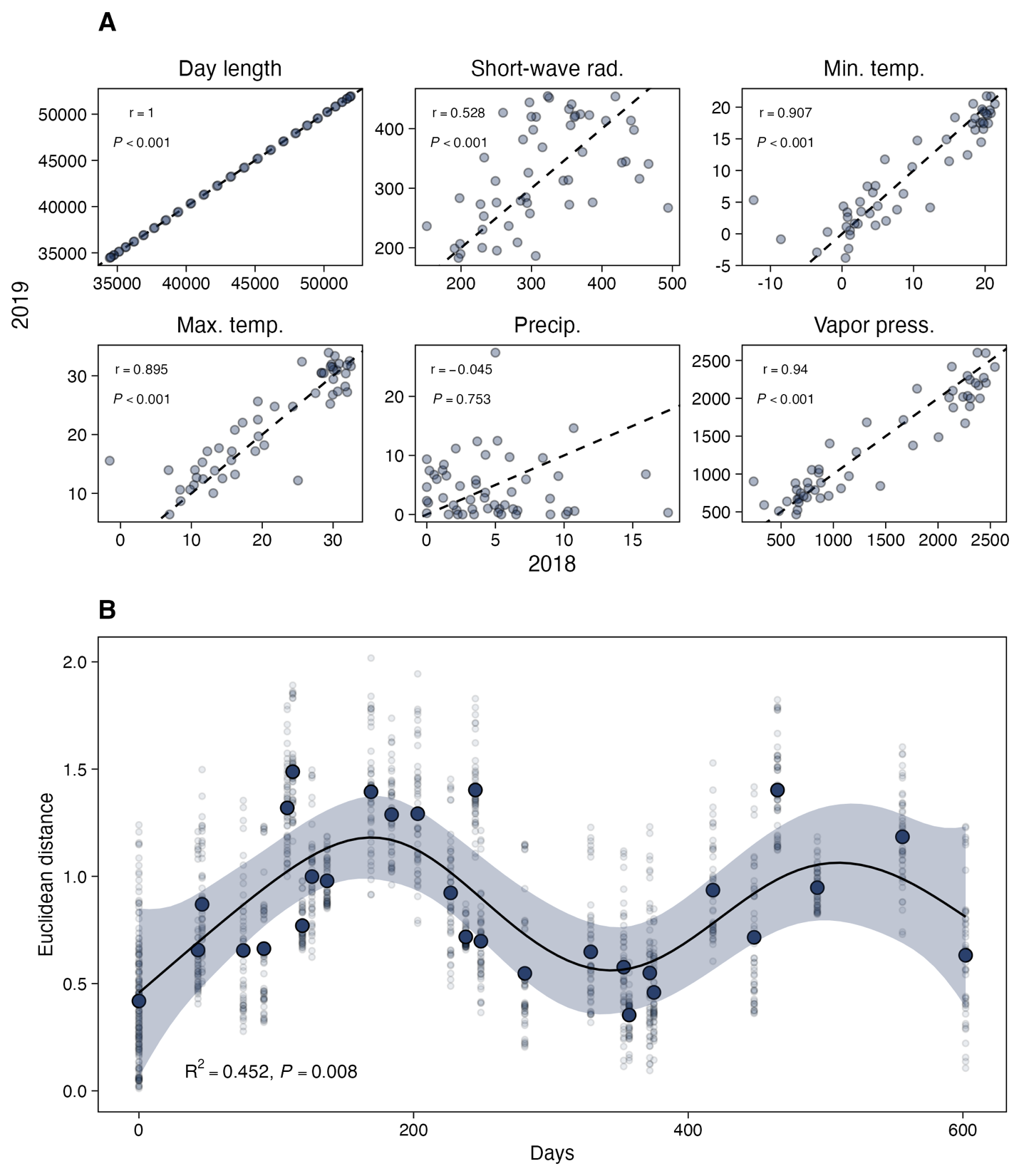


**Fig. S2:** Weekly mean climatic conditions were strongly correlated between years (**A**), with correlation coefficients and *P* values determined by Pearson correlations. Dashed lines represent 1:1 lines between weekly mean values in 2018 and 2019. Climatic dissimilarity oscillated with time between sample dates (**B**). Outlined points represent mean climatic dissimilarity and partially transparent points are individual distances (*n* $=$ 49 points per interval). Trend lines, R^2^ values, and *P* values were obtained from a generalized additive model (GAM) of mean values to avoid pseudo-replication.

**Table S1:** Results of three-way PERMANOVA tests (non-repeated measures) and two-way PERMANOVA tests (repeated measures) evaluating the difference in community composition between season, year, and host species (for three-way tests only). PERMANOVA was run on Bray-Curtis distance matrices calculated from ASV tables. Separate tests were run for each plant-associated habitat in bacterial/archaeal communities and fungal communities. Statistically significant values are in bold (*P* $<$ 0.05).

| **Plant habitat** | **Variable** | **Df** | **Sum of sqs.** | **R^2^** | **Pseudo-F** | **P** |
| --- | --- | --- | --- | --- | --- | --- |
| *Non-repeated measures* | |  |  |  |  |  |
| *Bacteria/Archaea* | |  |  |  |  |  |
| LE | **Host species** | **1** | **0.7** | **0.007** | **2.21** | **0.009** |
|  | **Year** | **1** | **7.1** | **0.077** | **23.8** | **< 0.001** |
|  | **Season** | **2** | **7.6** | **0.082** | **12.65** | **< 0.001** |
|  | **Host species x year** | **1** | **0.9** | **0.01** | **3.03** | **< 0.001** |
|  | Host species x season | 2 | 0.6 | 0.007 | 1.08 | 0.305 |
|  | **Year x season** | **2** | **4.6** | **0.05** | **7.72** | **< 0.001** |
|  | **Host species x year x season** | **2** | **0.9** | **0.01** | **1.52** | **0.036** |
|  | Residual | 234 | 70 | 0.757 |  |  |
|  | Total | 245 | 92.5 | 1 |  |  |
| RE | **Host species** | **1** | **1.2** | **0.009** | **3.39** | **< 0.001** |
|  | **Year** | **1** | **2.6** | **0.02** | **7.74** | **< 0.001** |
|  | **Season** | **3** | **4.9** | **0.037** | **4.79** | **< 0.001** |
|  | Host species x year | 1 | 0.4 | 0.003 | 1.16 | 0.158 |
|  | **Host species x season** | **3** | **1.2** | **0.009** | **1.18** | **0.046** |
|  | **Year x season** | **3** | **4.4** | **0.033** | **4.32** | **< 0.001** |
|  | Host species x year x season | 3 | 1.2 | 0.009 | 1.15 | 0.063 |
|  | Residual | 343 | 117.4 | 0.88 |  |  |
|  | Total | 358 | 133.4 | 1 |  |  |
| RH | **Host species** | **1** | **0.9** | **0.015** | **6.08** | **< 0.001** |
|  | **Year** | **1** | **1.5** | **0.027** | **10.66** | **< 0.001** |
|  | **Season** | **3** | **2.6** | **0.046** | **6.19** | **< 0.001** |
|  | Host species x year | 1 | 0.1 | 0.003 | 1.06 | 0.33 |
|  | Host species x season | 3 | 0.4 | 0.006 | 0.87 | 0.796 |
|  | **Year x season** | **3** | **2.6** | **0.046** | **6.14** | **< 0.001** |
|  | Host species x year x season | 3 | 0.4 | 0.008 | 1 | 0.445 |
|  | Residual | 340 | 47.7 | 0.849 |  |  |
|  | Total | 355 | 56.2 | 1 |  |  |
| *Fungi* | |  |  |  |  |  |
| LE | **Host species** | **1** | **1.1** | **0.013** | **4.18** | **< 0.001** |
|  | **Year** | **1** | **5.9** | **0.071** | **22.48** | **< 0.001** |
|  | **Season** | **2** | **7.2** | **0.087** | **13.69** | **< 0.001** |
|  | **Host species x year** | **1** | **1.3** | **0.016** | **4.89** | **< 0.001** |
|  | **Host species x season** | **2** | **0.9** | **0.011** | **1.66** | **0.02** |
|  | **Year x season** | **2** | **4.7** | **0.056** | **8.82** | **< 0.001** |

**Table S1, continued.**

| **Plant habitat** | **Variable** | **Df** | | **Sum of sqs.** | | **R^2^** | | **Pseudo-F** | | **P** | |
| --- | --- | --- | --- | --- | --- | --- | --- | --- | --- | --- | --- |
| *Fungi* | | |  | |  | |  | |  | |  |
| LE | **Host species x year x season** | **2** | | **1** | | **0.012** | | **1.84** | | **0.008** | |
|  | Residual | 232 | | 61.3 | | 0.735 | |  | |  | |
|  | Total | 243 | | 83.4 | | 1 | |  | |  | |
| RE | **Host species** | **1** | | **0.9** | | **0.008** | | **2.91** | | **< 0.001** | |
|  | **Year** | **1** | | **0.9** | | **0.009** | | **3.16** | | **< 0.001** | |
|  | **Season** | **3** | | **3.8** | | **0.035** | | **4.3** | | **< 0.001** | |
|  | Host species x year | 1 | | 0.2 | | 0.002 | | 0.6 | | 0.962 | |
|  | Host species x season | 3 | | 0.9 | | 0.008 | | 0.97 | | 0.514 | |
|  | **Year x season** | **3** | | **4.5** | | **0.041** | | **5.05** | | **< 0.001** | |
|  | Host species x year x season | 3 | | 0.9 | | 0.008 | | 1.04 | | 0.36 | |
|  | Residual | 330 | | 98.5 | | 0.89 | |  | |  | |
|  | Total | 345 | | 110.7 | | 1 | |  | |  | |
| RH | **Host species** | **1** | | **0.7** | | **0.008** | | **3.42** | | **< 0.001** | |
|  | **Year** | **1** | | **4.5** | | **0.048** | | **21.18** | | **< 0.001** | |
|  | **Season** | **3** | | **7.8** | | **0.083** | | **12.14** | | **< 0.001** | |
|  | Host species x year | 1 | | 0.3 | | 0.003 | | 1.39 | | 0.091 | |
|  | Host species x season | 3 | | 0.8 | | 0.009 | | 1.3 | | 0.055 | |
|  | **Year x season** | **3** | | **5** | | **0.054** | | **7.87** | | **< 0.001** | |
|  | Host species x year x season | 3 | | 0.8 | | 0.008 | | 1.23 | | 0.092 | |
|  | Residual | 344 | | 73.5 | | 0.786 | |  | |  | |
|  | Total | 359 | | 93.5 | | 1 | |  | |  | |
| *Repeated measures* | | |  | |  | |  | |  | |  |
| *Bacteria/Archaea* | | |  | |  | |  | |  | |  |
| LE | **Year** | **1** | | **7.1** | | **0.077** | | **23.34** | | **< 0.001** | |
|  | **Season** | **2** | | **7.5** | | **0.082** | | **12.35** | | **< 0.001** | |
|  | **Year x season** | **2** | | **4.5** | | **0.049** | | **7.42** | | **< 0.001** | |
|  | Residual | 240 | | 73.3 | | 0.792 | |  | |  | |
|  | Total | 245 | | 92.5 | | 1 | |  | |  | |
| RE | **Year** | **1** | | **2.7** | | **0.02** | | **7.67** | | **< 0.001** | |
|  | **Season** | **3** | | **4.9** | | **0.037** | | **4.75** | | **< 0.001** | |
|  | **Year x season** | **3** | | **4.4** | | **0.033** | | **4.28** | | **< 0.001** | |
|  | Residual | 351 | | 121.4 | | 0.91 | |  | |  | |
|  | Total | 358 | | 133.4 | | 1 | |  | |  | |
| RH | **Year** | **1** | | **1.5** | | **0.026** | | **10.46** | | **< 0.001** | |
|  | **Season** | **3** | | **2.6** | | **0.047** | | **6.16** | | **< 0.001** | |
|  | **Year x season** | **3** | | **2.6** | | **0.046** | | **6.07** | | **< 0.001** | |
|  | Residual | 348 | | 49.5 | | 0.881 | |  | |  | |
|  | Total | 355 | | 56.2 | | 1 | |  | |  | |
| *Fungi* | | |  | |  | |  | |  | |  |
| LE | **Year** | **1** | | **5.9** | | **0.071** | | **21.56** | | **< 0.001** | |
|  | **Season** | **2** | | **7.2** | | **0.087** | | **13.11** | | **< 0.001** | |

**Table S1, continued.**

| **Plant habitat** | **Variable** | **Df** | **Sum of sqs.** | **R^2^** | **Pseudo-F** | **P** |
| --- | --- | --- | --- | --- | --- | --- |
| *Fungi* | |  |  |  |  |  |
| LE | **Year x season** | **2** | **4.6** | **0.056** | **8.43** | **< 0.001** |
|  | Residual | 238 | 65.6 | 0.786 |  |  |
|  | Total | 243 | 83.4 | 1 |  |  |
| RE | **Year** | **1** | **1** | **0.009** | **3.17** | **< 0.001** |
|  | **Season** | **3** | **3.9** | **0.035** | **4.28** | **< 0.001** |
|  | **Year x season** | **3** | **4.5** | **0.041** | **5.04** | **< 0.001** |
|  | Residual | 338 | 101.3 | 0.916 |  |  |
|  | Total | 345 | 110.7 | 1 |  |  |
| RH | **Year** | **1** | **4.5** | **0.048** | **20.88** | **< 0.001** |
|  | **Season** | **3** | **7.8** | **0.083** | **11.98** | **< 0.001** |
|  | **Year x season** | **3** | **5.1** | **0.054** | **7.79** | **< 0.001** |
|  | Residual | 352 | 76.1 | 0.814 |  |  |
|  | Total | 359 | 93.5 | 1 |  |  |

**Table S2:** Results of three-way ANOVA tests evaluating the difference in community dispersion between seasons, year, and host species. Separate tests were run for each plant-associated habitat within bacterial/archaeal communities and fungal communities. Statistically significant values are in bold (*P* $<$ 0.05).

| **Plant habitat** | **Variable** | **Df** | **Sum of sqs.** | **F** | **P** |
| --- | --- | --- | --- | --- | --- |
| *Bacteria/Archaea* | |  |  |  |  |
| LE | **Year** | **1** | **0.1** | **7.39** | **0.007** |
|  | Season | 2 | 0.1 | 2.9 | 0.057 |
|  | **Host species** | **1** | **0.1** | **10.51** | **0.001** |
|  | Year x season | 2 | 0.1 | 2.27 | 0.106 |
|  | **Year x host species** | **1** | **0.2** | **12.39** | **< 0.001** |
|  | **Season x host species** | **2** | **0.1** | **3.15** | **0.045** |
|  | Year x season x host species | 2 | 0 | 1.35 | 0.26 |
|  | Residuals | 234 | 3.2 |  |  |
| RE | Year | 1 | 0 | 2.61 | 0.107 |
|  | **Season** | **3** | **0.1** | **7.58** | **< 0.001** |
|  | Host species | 1 | 0 | 0.88 | 0.349 |
|  | **Year x season** | **3** | **0.1** | **15.08** | **< 0.001** |
|  | Year x host species | 1 | 0 | 0.03 | 0.864 |
|  | Season x host species | 3 | 0 | 1.48 | 0.219 |
|  | Year x season x host species | 3 | 0 | 1.13 | 0.339 |
|  | Residuals | 343 | 1.1 |  |  |
| RH | Year | 1 | 0 | 2.27 | 0.133 |
|  | **Season** | **3** | **0** | **3.07** | **0.028** |
|  | Host species | 1 | 0 | 1.97 | 0.161 |
|  | **Year x season** | **3** | **0.1** | **5.93** | **< 0.001** |
|  | Year x host species | 1 | 0 | 0.21 | 0.647 |
|  | Season x host species | 3 | 0 | 0.92 | 0.429 |
|  | Year x season x host species | 3 | 0 | 1.18 | 0.319 |
|  | Residuals | 340 | 1.5 |  |  |
| *Fungi* | |  |  |  |  |
| LE | Year | 1 | 0 | 0.16 | 0.69 |
|  | **Season** | **2** | **0.1** | **4.5** | **0.012** |
|  | **Host species** | **1** | **0.2** | **15.25** | **< 0.001** |
|  | Year x season | 2 | 0 | 0.25 | 0.778 |
|  | **Year x host species** | **1** | **0.1** | **6.02** | **0.015** |
|  | Season x host species | 2 | 0 | 0.17 | 0.848 |
|  | Year x season x host species | 2 | 0 | 1.44 | 0.239 |
|  | Residuals | 232 | 3.2 |  |  |
| RE | Year | 1 | 0 | 2.16 | 0.143 |
|  | **Season** | **3** | **0.2** | **3.14** | **0.026** |
|  | **Host species** | **1** | **0.3** | **14.6** | **< 0.001** |
|  | **Year x season** | **3** | **0.6** | **8.31** | **< 0.001** |
|  | Year x host species | 1 | 0 | 0.23 | 0.633 |
|  | Season x host species | 3 | 0 | 0.08 | 0.97 |

**Table S2, continued.**

| *Fungi* | |  |  |  |  |
| --- | --- | --- | --- | --- | --- |
|  | Year x season x host species | 3 | 0 | 0.67 | 0.569 |
|  | Residuals | 330 | 7.3 |  |  |
| RH | Year | 1 | 0 | 0.24 | 0.621 |
|  | **Season** | **3** | **0.1** | **5.73** | **< 0.001** |
|  | Host species | 1 | 0 | 0.14 | 0.709 |
|  | Year x season | 3 | 0 | 1.12 | 0.34 |
|  | Year x host species | 1 | 0 | 0.18 | 0.675 |
|  | Season x host species | 3 | 0 | 0.68 | 0.566 |
|  | Year x season x host species | 3 | 0 | 0.6 | 0.616 |
|  | Residuals | 344 | 2.6 |  |  |

**Table S3:** Results of three-way ANOVA tests evaluating the difference in $\alpha$-diversity (^1^*D*; Hill number, q $=$ 0) between seasons, year, and host species. Separate tests were run for bacterial/archaeal communities and fungal communities within each plant-associated habitat. Statistically significant values are in bold (*P* $<$ 0.05). *, Did not remain significant with repeated-measures ANOVA (*P* = 0.08).

| **Hill index** | | **Variable** | | **Df** | | **Sum of sqs.** | | **F** | | **P** |
| --- | --- | --- | --- | --- | --- | --- | --- | --- | --- | --- |
| *Bacteria/Archaea* | | | |  | |  | |  | |  |
| *Leaf endosphere* | | | |  | |  | |  | |  |
| ^1^*D* | | Host species | | 1 | | 207.6 | | 2 | | 0.158 |
|  |  | **Year** | | **1** | | **1762.9** | | **17.02** | | **< 0.001** |
|  |  | **Season** | | **2** | | **1302.1** | | **6.28** | | **0.002** |
|  |  | Host species x year | | 1 | | 339 | | 3.27 | | 0.072 |
|  |  | **Host species x season** | | **2** | | **899.1** | | **4.34** | | **0.014** |
|  |  | **Year x season** | | **2** | | **4336.9** | | **20.93** | | **< 0.001** |
|  |  | Host species x year x season | | 2 | | 199.7 | | 0.96 | | 0.383 |
|  |  | Residuals | | 234 | | 24244.4 | |  | |  |
| *Root endosphere* | | | |  | |  | |  | |  |
| ^1^*D* | | Host species | | 1 | | 18 | | 0.01 | | 0.941 |
|  |  | Year | | 1 | | 4512.6 | | 1.35 | | 0.245 |
|  |  | **Season** | | **3** | | **99333.4** | | **9.94** | | **< 0.001** |
|  |  | Host species x year | | 1 | | 1078.6 | | 0.32 | | 0.57 |
|  |  | **Host species x season** | | **3** | | **27743.4** | | **2.78** | | **0.041** |
|  |  | **Year x season** | | **3** | | **91630.2** | | **9.17** | | **< 0.001** |
|  |  | **Host species x year x season** | | **3** | | **37485.3** | | **3.75** | | **0.011** |
|  |  | Residuals | | 343 | | 1142462 | |  | |  |
| *Rhizosphere* | | | |  | |  | |  | |  |
| ^1^*D* | | Host species | | 1 | | 112428.8 | | 3.1 | | 0.079 |
|  |  | **Year** | | **1** | | **1219660.9** | | **33.6** | | **< 0.001** |
|  |  | **Season** | | **3** | | **463882.9** | | **4.26** | | **0.006** |
|  |  | Host species x year | | 1 | | 3822.6 | | 0.11 | | 0.746 |
|  |  | Host species x season | | 3 | | 62617.2 | | 0.58 | | 0.632 |
|  |  | **Year x season** | | **3** | | **1098080** | | **10.08** | | **< 0.001** |
|  |  | **Host species x year x season** | | **3** | | **374815.3** | | **3.44** | | **0.017** |
|  |  | Residuals | | 340 | | 12341489 | |  | |  |
| *Fungi* | | |  | |  | |  | |  | |
| *Leaf endosphere* | | |  | |  | |  | |  | |
| ^0^*D* | Host species | | 1 | | 945.8 | | 2.75 | | 0.099 | |
|  | **Year** | | **1** | | **17339.8** | | **50.32** | | **< 0.001** | |
|  | **Season** | | **2** | | **11280.8** | | **16.37** | | **< 0.001** | |
|  | Host species x year | | 1 | | 8.3 | | 0.02 | | 0.877 | |
|  | Host species x season | | 2 | | 884.2 | | 1.28 | | 0.279 | |
|  | **Year x season** | | **2** | | **10085.1** | | **14.63** | | **< 0.001** | |
|  | Host species x year x season | | 2 | | 1207.3 | | 1.75 | | 0.176 | |
|  | Residuals | | 232 | | 79938.5 | |  | |  | |

**Table S3, continued.**

| **Hill index** | **Variable** | **Df** | **Sum of sqs.** | **F** | **P** |
| --- | --- | --- | --- | --- | --- |
| *Fungi* | |  |  |  |  |
| *Root endosphere* | |  |  |  |  |
| ^1^*D* | **Host species** | **1** | **80.1** | **4.31** | **0.039** |
|  | **Year** | **1** | **347.3** | **18.69** | **< 0.001** |
|  | **Season** | **3** | **202.3** | **3.63** | **0.013** |
|  | Host species x year | 1 | 3.3 | 0.18 | 0.673 |
|  | Host species x season | 3 | 43.8 | 0.79 | 0.503 |
|  | **Year x season** | **3** | **1147.1** | **20.57** | **< 0.001*** |
|  | Host species x year x season | 3 | 38.8 | 0.7 | 0.555 |
|  | Residuals | 330 | 6132.6 |  |  |
| *Rhizosphere* | |  |  |  |  |
| ^1^*D* | Host species | 1 | 2058.9 | 2.95 | 0.087 |
|  | **Year** | **1** | **13285.5** | **19.04** | **< 0.001** |
|  | **Season** | **3** | **17397.7** | **8.31** | **< 0.001** |
|  | Host species x year | 1 | 427.9 | 0.61 | 0.434 |
|  | Host species x season | 3 | 2103.9 | 1 | 0.391 |
|  | Year x season | 3 | 4851.8 | 2.32 | 0.075 |
|  | Host species x year x season | 3 | 709.9 | 0.34 | 0.797 |
|  | Residuals | 344 | 240064.3 |  |  |

**Table S4:** Percentage of sequences within each core group belonging to microbial genera.

| **Plant habitat** | **Phylum** | **Genus** | **Group** | **% of group** |
| --- | --- | --- | --- | --- |
| *Bacteria and Archaea* | |  |  |  |
| LE | Proteobacteria | *Sphingomonas* | 1 | 34 |
|  | Proteobacteria | *Methylob.-Methylor.* | 1 | 21 |
|  | Proteobacteria | *Pseudomonas* | 1 | 17 |
|  | Firmicutes | *Anaerobacillus* | 2 | 46 |
|  | Proteobacteria | *Escherichia-Shigella* | 2 | 18 |
|  | Firmicutes | *Staphylococcus* | 2 | 16 |
|  | Firmicutes | *Bacillus* | 2 | 14 |
|  | Actinobacteriota | *Cutibacterium* | 3 | 30 |
|  | Proteobacteria | *Acinetobacter* | 3 | 25 |
|  | Proteobacteria | *Pseudomonas* | 3 | 24 |
|  | Proteobacteria | *Halomonas* | 3 | 22 |
|  | Proteobacteria | *Pseudomonas* | 4 | 100 |
| RE | Actinobacteriota | Uncl. Pseudonocardiaceae | 1 | 47 |
|  | Actinobacteriota | *Actinophytocola* | 1 | 38 |
|  | Proteobacteria | *Allo.-Neo.-Para.-Rhizo.* | 2 | 46 |
|  | Proteobacteria | Uncl. Comamonadaceae | 2 | 12 |
|  | Proteobacteria | *Novosphingobium* | 2 | 11 |
|  | Proteobacteria | *Bradyrhizobium* | 3 | 29 |
|  | Actinobacteriota | Uncl. Micromonosporaceae | 3 | 23 |
|  | Proteobacteria | *Rhodomicrobium* | 3 | 21 |
|  | Proteobacteria | Uncl. Comamonadaceae | 3 | 11 |
|  | Firmicutes | *Anaerobacillus* | 4 | 40 |
|  | Firmicutes | *Bacillus* | 4 | 15 |
|  | Proteobacteria | *Escherichia-Shigella* | 4 | 13 |
|  | Actinobacteriota | *Cutibacterium* | 4 | 12 |
|  | Actinobacteriota | *Streptomyces* | 5 | 60 |
|  | Proteobacteria | *Acinetobacter* | 5 | 11 |
| RH | Crenarchaeota | Uncl. Nitrososphaeraceae | 1 | 36 |
|  | Actinobacteriota | Uncl. Gaiellales | 2 | 13 |
|  | Verrucomicrobiota | *Candidatus Udaeobacter* | 2 | 12 |
|  | Crenarchaeota | Uncl. Nitrososphaeraceae | 3 | 21 |
|  | Firmicutes | *Bacillus* | 3 | 13 |
|  | Crenarchaeota | Uncl. Nitrososphaeraceae | 4 | 22 |
| *Fungi* | |  |  |  |
| LE | Ascomycota | Uncl. Pleosporales | 1 | 42 |
|  | Ascomycota | *Cercospora* | 1 | 32 |
|  | Ascomycota | Uncl. Dothideomycetes | 1 | 14 |
|  | Ascomycota | *Rachicladosporium* | 1 | 12 |
|  | Ascomycota | Uncl. Pleosporales | 2 | 100 |
|  | Ascomycota | *Cladosporium* | 3 | 44 |
|  | Basidiomycota | Uncl. Inocybaceae | 3 | 40 |
|  | Ascomycota | *Exophiala* | 4 | 29 |

**Table S4, continued.**

| **Plant habitat** | **Phylum** | **Genus** | **Group** | **% of group** |
| --- | --- | --- | --- | --- |
| *Bacteria and Archaea* | |  |  |  |
| LE | Ascomycota | Uncl. Nectriaceae | 4 | 24 |
|  | Ascomycota | *Fusarium* | 4 | 24 |
|  | Ascomycota | *Plectosphaerella* | 4 | 23 |
|  | Ascomycota | Uncl. Ascomycota | 5 | 61 |
|  | Ascomycota | *Alternaria* | 5 | 39 |
| RE | Ascomycota | Uncl. Pleosporales | 1 | 87 |
|  | Ascomycota | Uncl. Helotiaceae | 2 | 56 |
|  | Ascomycota | Uncl. Pyronemataceae | 2 | 44 |
| RH | Ascomycota | *Cladosporium* | 1 | 35 |
|  | Ascomycota | Uncl. Sordariomycetes | 1 | 35 |
|  | Basidiomycota | Uncl. Inocybaceae | 2 | 58 |
|  | Ascomycota | *Cladosporium* | 2 | 13 |
|  | Mortierellomycota | *Mortierella* | 3 | 26 |
|  | Ascomycota | Uncl. Pleosporales | 3 | 10 |
|  | Ascomycota | Uncl. Hypocreales | 4 | 41 |
|  | Ascomycota | Uncl. Pleosporales | 4 | 40 |
|  | Ascomycota | Uncl. Pleosporales | 5 | 17 |
|  | Ascomycota | *Alternaria* | 5 | 12 |

*Methylob.-Methylor., Methylobacterium-Methylorubrum*

*Allo.-Neo.-Para.-Rhizo., Allorhizobium-Neorhizobium-Pararhizobium-Rhizobium*

**Table S5:** Results of three-way ANOVA tests evaluating the difference in the relative abundance of select core groups between seasons, year, and host species. Separate tests were run for each group within bacterial/archaeal communities and fungal communities by plant-associated habitat. Statistically significant values are in bold (*P* $<$ 0.05).

| **Group** | **Variable** | **Df** | **Sum of sqs.** | **F** | **P** |
| --- | --- | --- | --- | --- | --- |
| *Bacteria and Archaea* | |  |  |  |  |
| *Leaf endosphere* | |  |  |  |  |
| Group 1 | **Year** | **1** | **2.6** | **82.9** | **< 0.001** |
| Group 1 | **Season** | **2** | **4.2** | **65.23** | **< 0.001** |
| Group 1 | **Host species** | **1** | **0.1** | **3.94** | **0.048** |
| Group 1 | **Year x season** | **2** | **2.4** | **38.42** | **< 0.001** |
| Group 1 | **Year x host species** | **1** | **0.4** | **12.58** | **< 0.001** |
| Group 1 | Season x host species | 2 | 0 | 0.68 | 0.508 |
| Group 1 | Year x season x host species | 2 | 0.2 | 2.59 | 0.077 |
| Group 1 | Residuals | 234 | 7.4 |  |  |
| Group 4 | **Year** | **1** | **3.1** | **64.7** | **< 0.001** |
| Group 4 | **Season** | **2** | **0.9** | **9.34** | **< 0.001** |
| Group 4 | Host species | 1 | 0.1 | 1.16 | 0.282 |
| Group 4 | **Year x season** | **2** | **0.4** | **4.72** | **0.010** |
| Group 4 | Year x host species | 1 | 0 | 0.16 | 0.685 |
| Group 4 | Season x host species | 2 | 0 | 0.2 | 0.818 |
| Group 4 | Year x season x host species | 2 | 0 | 0.06 | 0.939 |
| Group 4 | Residuals | 234 | 11 |  |  |
| *Root endosphere* | |  |  |  |  |
| Group 1 | **Year** | **1** | **0.3** | **36.01** | **< 0.001** |
| Group 1 | **Season** | **3** | **0.1** | **3.54** | **0.015** |
| Group 1 | **Host species** | **1** | **0** | **4.18** | **0.042** |
| Group 1 | **Year x season** | **3** | **0.1** | **3.62** | **0.013** |
| Group 1 | **Year x host species** | **1** | **0** | **4.97** | **0.026** |
| Group 1 | **Season x host species** | **3** | **0.1** | **4.57** | **0.004** |
| Group 1 | Year x season x host species | 3 | 0 | 1.7 | 0.167 |
| Group 1 | Residuals | 343 | 3 |  |  |
| Group 2 | Year | 1 | 0 | 0.34 | 0.559 |
| Group 2 | **Season** | **3** | **0.1** | **5.18** | **0.002** |
| Group 2 | Host species | 1 | 0 | 0 | 0.982 |
| Group 2 | Year x season | 3 | 0 | 1.65 | 0.178 |
| Group 2 | Year x host species | 1 | 0 | 1.03 | 0.311 |
| Group 2 | Season x host species | 3 | 0.1 | 2.2 | 0.088 |
| Group 2 | Year x season x host species | 3 | 0 | 1.14 | 0.333 |
| Group 2 | Residuals | 343 | 3.3 |  |  |
| Group 3 | **Year** | **1** | **0.1** | **13.43** | **< 0.001** |
| Group 3 | **Season** | **3** | **0.1** | **6.94** | **< 0.001** |
| Group 3 | Host species | 1 | 0 | 1.34 | 0.247 |
| Group 3 | **Year x season** | **3** | **0.2** | **11.54** | **< 0.001** |
| Group 3 | Year x host species | 1 | 0 | 0.2 | 0.659 |

**Table S5, continued.**

| **Group** | **Variable** | **Df** | **Sum of sqs.** | **F** | **P** |
| --- | --- | --- | --- | --- | --- |
| *Bacteria and Archaea* | |  |  |  |  |
| *Root endosphere* | |  |  |  |  |
| Group 3 | Season x host species | 3 | 0 | 0.03 | 0.994 |
| Group 3 | Year x season x host species | 3 | 0 | 0.47 | 0.703 |
| Group 3 | Residuals | 343 | 1.5 |  |  |
| Group 4 | **Year** | **1** | **0.3** | **53.28** | **< 0.001** |
| Group 4 | **Season** | **3** | **0.4** | **21.99** | **< 0.001** |
| Group 4 | Host species | 1 | 0 | 0.83 | 0.362 |
| Group 4 | **Year x season** | **3** | **0.2** | **11.81** | **< 0.001** |
| Group 4 | Year x host species | 1 | 0 | 0.04 | 0.835 |
| Group 4 | Season x host species | 3 | 0 | 0.46 | 0.710 |
| Group 4 | Year x season x host species | 3 | 0 | 0.18 | 0.907 |
| Group 4 | Residuals | 343 | 2.1 |  |  |
| Group 5 | Year | 1 | 0 | 0.31 | 0.581 |
| Group 5 | **Season** | **3** | **0.2** | **9.2** | **< 0.001** |
| Group 5 | **Host species** | **1** | **0.2** | **23.86** | **< 0.001** |
| Group 5 | Year x season | 3 | 0 | 0.85 | 0.470 |
| Group 5 | Year x host species | 1 | 0 | 2.25 | 0.135 |
| Group 5 | **Season x host species** | **3** | **0.1** | **3.21** | **0.023** |
| Group 5 | Year x season x host species | 3 | 0 | 1.3 | 0.273 |
| Group 5 | Residuals | 343 | 2.7 |  |  |
| *Rhizosphere* | |  |  |  |  |
| Group 1 | **Year** | **1** | **0.1** | **32.1** | **< 0.001** |
| Group 1 | **Season** | **3** | **0** | **4.37** | **0.005** |
| Group 1 | **Host species** | **1** | **0** | **14.92** | **< 0.001** |
| Group 1 | Year x season | 3 | 0 | 1.29 | 0.277 |
| Group 1 | Year x host species | 1 | 0 | 0.14 | 0.707 |
| Group 1 | Season x host species | 3 | 0 | 0.55 | 0.648 |
| Group 1 | Year x season x host species | 3 | 0 | 1.57 | 0.198 |
| Group 1 | Residuals | 340 | 1.1 |  |  |
| Group 2 | **Year** | **1** | **0** | **3.98** | **0.047** |
| Group 2 | **Season** | **3** | **0** | **6.8** | **< 0.001** |
| Group 2 | Host species | 1 | 0 | 1.57 | 0.210 |
| Group 2 | **Year x season** | **3** | **0** | **9.43** | **< 0.001** |
| Group 2 | Year x host species | 1 | 0 | 0.04 | 0.835 |
| Group 2 | Season x host species | 3 | 0 | 0.13 | 0.941 |
| Group 2 | Year x season x host species | 3 | 0 | 0.03 | 0.993 |
| Group 2 | Residuals | 340 | 0.4 |  |  |
| Group 3 | **Year** | **1** | **0.1** | **19.25** | **< 0.001** |
| Group 3 | **Season** | **3** | **0** | **4.33** | **0.005** |
| Group 3 | **Host species** | **1** | **0** | **13.15** | **< 0.001** |
| Group 3 | **Year x season** | **3** | **0.2** | **30.15** | **< 0.001** |
| Group 3 | Year x host species | 1 | 0 | 0.11 | 0.744 |

**Table S5, continued.**

| **Group** | **Variable** | **Df** | **Sum of sqs.** | **F** | **P** |
| --- | --- | --- | --- | --- | --- |
| *Bacteria and Archaea* | |  |  |  |  |
| *Rhizosphere* | |  |  |  |  |
| Group 3 | Season x host species | 3 | 0 | 0.01 | 0.999 |
| Group 3 | Year x season x host species | 3 | 0 | 0.81 | 0.490 |
| Group 3 | Residuals | 340 | 0.9 |  |  |
| Group 4 | **Year** | **1** | **0** | **20.38** | **< 0.001** |
| Group 4 | **Season** | **3** | **0** | **4.49** | **0.004** |
| Group 4 | **Host species** | **1** | **0** | **11.11** | **< 0.001** |
| Group 4 | **Year x season** | **3** | **0.1** | **17.64** | **< 0.001** |
| Group 4 | Year x host species | 1 | 0 | 1.34 | 0.247 |
| Group 4 | Season x host species | 3 | 0 | 0.48 | 0.700 |
| Group 4 | Year x season x host species | 3 | 0 | 1.9 | 0.129 |
| Group 4 | Residuals | 340 | 0.5 |  |  |
| *Fungi* | |  |  |  |  |
| *Leaf endosphere* | |  |  |  |  |
| Group 2 | **Year** | **1** | **0.9** | **25.49** | **< 0.001** |
| Group 2 | **Season** | **2** | **1.6** | **22.82** | **< 0.001** |
| Group 2 | Host species | 1 | 0.1 | 3.06 | 0.081 |
| Group 2 | **Year x season** | **2** | **0.4** | **6.18** | **0.002** |
| Group 2 | Year x host species | 1 | 0.1 | 1.54 | 0.216 |
| Group 2 | Season x host species | 2 | 0 | 0.51 | 0.600 |
| Group 2 | Year x season x host species | 2 | 0 | 0.63 | 0.533 |
| Group 2 | Residuals | 231 | 8.2 |  |  |
| Group 5 | **Year** | **1** | **1** | **38.05** | **< 0.001** |
| Group 5 | **Season** | **2** | **1.7** | **32.72** | **< 0.001** |
| Group 5 | **Host species** | **1** | **0.2** | **8.69** | **0.004** |
| Group 5 | **Year x season** | **2** | **1.2** | **21.98** | **< 0.001** |
| Group 5 | **Year x host species** | **1** | **0.2** | **9.01** | **0.003** |
| Group 5 | **Season x host species** | **2** | **0.3** | **4.72** | **0.010** |
| Group 5 | Year x season x host species | 2 | 0.1 | 1.06 | 0.349 |
| Group 5 | Residuals | 231 | 6.1 |  |  |
| *Root endosphere* | |  |  |  |  |
| Group 2 | **Year** | **1** | **0.1** | **4.39** | **0.037** |
| Group 2 | **Season** | **3** | **1.3** | **17.69** | **< 0.001** |
| Group 2 | Host species | 1 | 0 | 0.4 | 0.529 |
| Group 2 | **Year x season** | **3** | **0.7** | **9.04** | **< 0.001** |
| Group 2 | Year x host species | 1 | 0 | 0.2 | 0.656 |
| Group 2 | Season x host species | 3 | 0 | 0.31 | 0.815 |
| Group 2 | Year x season x host species | 3 | 0 | 0.22 | 0.883 |
| Group 2 | Residuals | 322 | 7.8 |  |  |
| *Rhizosphere* | |  |  |  |  |
| Group 2 | **Year** | **1** | **1.5** | **71.89** | **< 0.001** |
| Group 2 | **Season** | **3** | **1.8** | **27.99** | **< 0.001** |

**Table S5, continued.**

| **Group** | **Variable** | **Df** | **Sum of sqs.** | **F** | **P** |
| --- | --- | --- | --- | --- | --- |
| *Fungi* | |  |  |  |  |
| *Rhizosphere* | |  |  |  |  |
| Group 2 | Host species | 1 | 0 | 1.97 | 0.162 |
| Group 2 | Year x season | 3 | 0.1 | 1.15 | 0.330 |
| Group 2 | Year x host species | 1 | 0 | 0.36 | 0.546 |
| Group 2 | Season x host species | 3 | 0 | 0.26 | 0.852 |
| Group 2 | Year x season x host species | 3 | 0.1 | 1.79 | 0.149 |
| Group 2 | Residuals | 344 | 7.3 |  |  |
| Group 3 | **Year** | **1** | **0.7** | **60.55** | **< 0.001** |
| Group 3 | **Season** | **3** | **1.1** | **29.8** | **< 0.001** |
| Group 3 | Host species | 1 | 0 | 2.01 | 0.157 |
| Group 3 | **Year x season** | **3** | **0.3** | **7.73** | **< 0.001** |
| Group 3 | Year x host species | 1 | 0 | 2.49 | 0.116 |
| Group 3 | Season x host species | 3 | 0 | 0.15 | 0.929 |
| Group 3 | Year x season x host species | 3 | 0 | 0.72 | 0.539 |
| Group 3 | Residuals | 344 | 4 |  |  |

**Table S6:** Characteristics of co-occurrence networks for leaf endosphere, root endosphere, and rhizosphere microbial communities.

| **Network characteristic** | **Leaf endosphere** | **Root endosphere** | **Rhizosphere** |
| --- | --- | --- | --- |
| Nodes | 115 | 156 | 2536 |
| Nodes with edges | 41 | 27 | 2319 |
| Edges | 38 | 17 | 9027 |
| Positive edges | 27 | 17 | 7684 |
| Negative edges | 11 | 0 | 1343 |
| Density | 0.0058 | 0.0014 | 0.0028 |
| Degree | 0.661 [0.379, 0.943] | 0.218 [0.126, 0.310] | 7.119 [6.837, 7.402] |
| Clustering coefficient | 0.15 | 0.231 | 0.103 |
| Heterogeneity | 0.357 [0.268, 0.445] | 0.173 [0.113, 0.233] | 0.914 [0.904, 0.925] |
| Mean shortest path | 0.198 | 0.044 | 0.076 |
| Hub ASVs | 11 | 3 | 107 |

**Table S7:** Genus-level taxonomic classifications of hub ASVs.

| **ASV** | **Domain or kingdom** | **Genus** | **Degree** | **Betw.** |
| --- | --- | --- | --- | --- |
| *Leaf endosphere* | |  |  |  |
| Bac./Arc. ASV 319 | Bacteria | *Methylob.-Methylor.* | 13 | 160 |
| Bac./Arc. ASV 258 | Bacteria | *Methylob.-Methylor.* | 5 | 64 |
| Bac./Arc. ASV 122 | Bacteria | *Sphingomonas* | 4 | 69 |
| Bac./Arc. ASV 40 | Bacteria | *Bacillus* | 4 | 53 |
| Bac./Arc. ASV 85 | Bacteria | *Pseudomonas* | 4 | 19 |
| Fun. ASV 1 | Fungi | Uncl. Inocybaceae | 4 | 6 |
| Fun. ASV 81 | Fungi | *Exophiala* | 3 | 3 |
| Bac./Arc. ASV 229 | Bacteria | *Roseomonas* | 2 | 75 |
| Bac./Arc. ASV 143 | Bacteria | *Sphingomonas* | 2 | 19 |
| Fun. ASV 14 | Fungi | *Alternaria* | 2 | 1 |
| *Root endosphere* | |  |  |  |
| Bac./Arc. ASV 50 | Bacteria | *Cutibacterium* | 5 | 9 |
| Bac./Arc. ASV 79 | Bacteria | *Haliangium* | 2 | 1 |
| *Rhizosphere* | |  |  |  |
| Bac./Arc. ASV 7 | Archaea | Uncl. Nitrososphaeraceae | 58 | 401134 |
| Bac./Arc. ASV 9 | Archaea | Uncl. Nitrososphaeraceae | 56 | 46587 |
| Bac./Arc. ASV 96 | Bacteria | *Nocardioides* | 50 | 167549 |
| Bac./Arc. ASV 182 | Archaea | Uncl. Nitrososphaeraceae | 47 | 30027 |
| Bac./Arc. ASV 1 | Archaea | Uncl. Nitrososphaeraceae | 40 | 256124 |
| Bac./Arc. ASV 4 | Bacteria | *Streptomyces* | 40 | 65310 |
| Bac./Arc. ASV 36 | Bacteria | Uncl. Vicinamibacterales | 37 | 277525 |
| Bac./Arc. ASV 2 | Archaea | Uncl. Nitrososphaeraceae | 36 | 33061 |
| Bac./Arc. ASV 29 | Bacteria | Uncl. Xanthobacteraceae | 35 | 32841 |
| Bac./Arc. ASV 13 | Archaea | Uncl. Nitrososphaeraceae | 30 | 87965 |
| Bac./Arc. ASV 25 | Bacteria | *Gaiella* | 30 | 58790 |
| Bac./Arc. ASV 43 | Bacteria | *Microvirga* | 25 | 127867 |
| Bac./Arc. ASV 112 | Bacteria | Uncl. Chitinophagaceae | 25 | 42721 |
| Bac./Arc. ASV 3 | Archaea | Uncl. Nitrososphaeraceae | 23 | 102173 |
| Bac./Arc. ASV 115 | Bacteria | JG30-KF-CM45 | 23 | 85311 |
| Bac./Arc. ASV 107 | Bacteria | Uncl. Vicinamibacteraceae | 23 | 59359 |
| Bac./Arc. ASV 105 | Bacteria | *Mesorhizobium* | 22 | 92653 |
| Fun. ASV 6 | Fungi | Uncl. Pleosporales | 22 | 36739 |
| Bac./Arc. ASV 8 | Bacteria | *Bacillus* | 20 | 40009 |
| Bac./Arc. ASV 24 | Bacteria | Uncl. Gaiellales | 20 | 24952 |
| Bac./Arc. ASV 42 | Bacteria | *Devosia* | 18 | 33519 |
| Fun. ASV 80 | Fungi | Uncl. Didymellaceae | 16 | 28321 |
| Bac./Arc. ASV 20 | Bacteria | Uncl. Vicinamibacteraceae | 16 | 28005 |

**Table S8:** Results of three-way ANOVA tests evaluating the difference in the relative abundance of fungal guilds between seasons, year, and host species. Separate tests were run for each guild within each plant-associated habitat. Statistically significant values are in bold (*P* $<$ 0.05).

| **Guild** | **Variable** | **Df** | **Sum of sqs.** | **F** | **P** |
| --- | --- | --- | --- | --- | --- |
| *Leaf endosphere* | |  |  |  |  |
| AMF | Year | 1 | 0.000 | 2.6 | 0.108 |
|  | Month | 2 | 0.000 | 0.69 | 0.503 |
|  | Host species | 1 | 0.000 | 0.03 | 0.855 |
|  | Year x month | 2 | 0.000 | 0.58 | 0.563 |
|  | Year x host species | 1 | 0.000 | 0.02 | 0.899 |
|  | Month x host species | 2 | 0.000 | 0.64 | 0.529 |
|  | Year x month x host species | 2 | 0.000 | 0.42 | 0.659 |
|  | Residuals | 232 | 0.000 |  |  |
| ECM | **Year** | **1** | **0.178** | **102.48** | **< 0.001** |
|  | **Month** | **2** | **0.054** | **15.49** | **< 0.001** |
|  | **Host species** | **1** | **0.012** | **6.83** | **0.01** |
|  | **Year x month** | **2** | **0.025** | **7.35** | **< 0.001** |
|  | Year x host species | 1 | 0.005 | 2.74 | 0.099 |
|  | **Month x host species** | **2** | **0.018** | **5.26** | **0.006** |
|  | **Year x month x host species** | **2** | **0.018** | **5.23** | **0.006** |
|  | Residuals | 232 | 0.402 |  |  |
| Endophyte | Year | 1 | 0.003 | 1.67 | 0.198 |
|  | Month | 2 | 0.002 | 0.54 | 0.581 |
|  | **Host species** | **1** | **0.009** | **4.81** | **0.029** |
|  | Year x month | 2 | 0.004 | 1.11 | 0.332 |
|  | Year x host species | 1 | 0.004 | 2.38 | 0.124 |
|  | Month x host species | 2 | 0.001 | 0.24 | 0.786 |
|  | **Year x month x host species** | **2** | **0.014** | **3.99** | **0.02** |
|  | Residuals | 232 | 0.411 |  |  |
| Plant Pathogen | Year | 1 | 0.003 | 0.42 | 0.519 |
|  | Month | 2 | 0.003 | 0.22 | 0.806 |
|  | Host species | 1 | 0.000 | 0 | 0.978 |
|  | Year x month | 2 | 0.033 | 2.2 | 0.113 |
|  | Year x host species | 1 | 0.025 | 3.32 | 0.07 |
|  | Month x host species | 2 | 0.027 | 1.84 | 0.161 |
|  | Year x month x host species | 2 | 0.017 | 1.11 | 0.33 |
|  | Residuals | 232 | 1.721 |  |  |
| Saprotroph | Year | 1 | 0.007 | 2.29 | 0.132 |
|  | **Month** | **2** | **0.168** | **27.21** | **< 0.001** |
|  | Host species | 1 | 0.001 | 0.21 | 0.645 |
|  | **Year x month** | **2** | **0.024** | **3.87** | **0.022** |
|  | Year x host species | 1 | 0.001 | 0.37 | 0.545 |
|  | Month x host species | 2 | 0.003 | 0.53 | 0.587 |

**Table S8, continued.**

| **Guild** | **Variable** | **Df** | **Sum of sqs.** | **F** | **P** |
| --- | --- | --- | --- | --- | --- |
| *Leaf endosphere* | |  |  |  |  |
| Saprotroph | Year x month x host species | 2 | 0.016 | 2.59 | 0.077 |
|  | Residuals | 232 | 0.714 |  |  |
| Unclassified | Year | 1 | 0.092 | 2.39 | 0.124 |
|  | **Month** | **2** | **1.384** | **17.9** | **< 0.001** |
|  | Host species | 1 | 0.002 | 0.05 | 0.829 |
|  | Year x month | 2 | 0.046 | 0.6 | 0.552 |
|  | Year x host species | 1 | 0.036 | 0.93 | 0.335 |
|  | Month x host species | 2 | 0.018 | 0.24 | 0.789 |
|  | Year x month x host species | 2 | 0.101 | 1.31 | 0.273 |
|  | Residuals | 232 | 8.972 |  |  |
| *Root endosphere* | |  |  |  |  |
| AMF | Year | 1 | 0.000 | 1.04 | 0.309 |
|  | Month | 3 | 0.001 | 1.56 | 0.200 |
|  | Host species | 1 | 0.000 | 0.36 | 0.548 |
|  | Year x month | 3 | 0.002 | 2.53 | 0.057 |
|  | Year x host species | 1 | 0.000 | 0.08 | 0.776 |
|  | Month x host species | 3 | 0.000 | 0.4 | 0.756 |
|  | Year x month x host species | 3 | 0.000 | 0.33 | 0.802 |
|  | Residuals | 330 | 0.065 |  |  |
| ECM | **Year** | **1** | **0.036** | **12.38** | **< 0.001** |
|  | **Month** | **3** | **0.194** | **22.45** | **< 0.001** |
|  | Host species | 1 | 0.002 | 0.81 | 0.367 |
|  | **Year x month** | **3** | **0.145** | **16.75** | **< 0.001** |
|  | Year x host species | 1 | 0.003 | 0.96 | 0.329 |
|  | Month x host species | 3 | 0.003 | 0.32 | 0.814 |
|  | Year x month x host species | 3 | 0.005 | 0.6 | 0.618 |
|  | Residuals | 330 | 0.953 |  |  |
| Endophyte | Year | 1 | 0.000 | 0.28 | 0.594 |
|  | **Month** | **3** | **0.017** | **3.68** | **0.012** |
|  | Host species | 1 | 0.001 | 0.69 | 0.405 |
|  | Year x month | 3 | 0.006 | 1.36 | 0.253 |
|  | Year x host species | 1 | 0.000 | 0 | 0.957 |
|  | Month x host species | 3 | 0.006 | 1.16 | 0.324 |
|  | Year x month x host species | 3 | 0.004 | 0.94 | 0.421 |
|  | Residuals | 330 | 0.521 |  |  |
| Plant Pathogen | Year | 1 | 0.033 | 2.85 | 0.092 |
|  | Month | 3 | 0.086 | 2.46 | 0.062 |
|  | Host species | 1 | 0.001 | 0.04 | 0.835 |
|  | **Year x month** | **3** | **0.314** | **9** | **< 0.001** |
|  | Year x host species | 1 | 0.000 | 0.02 | 0.886 |
|  | Month x host species | 3 | 0.012 | 0.36 | 0.785 |

**Table S8, continued.**

| **Guild** | **Variable** | **Df** | **Sum of sqs.** | **F** | **P** |
| --- | --- | --- | --- | --- | --- |
| *Root endosphere* | |  |  |  |  |
| Plant Pathogen | Year x month x host species | 3 | 0.034 | 0.97 | 0.407 |
|  | Residuals | 330 | 3.834 |  |  |
| Saprotroph | Year | 1 | 0.007 | 0.23 | 0.632 |
|  | **Month** | **3** | **0.254** | **2.74** | **0.043** |
|  | Host species | 1 | 0.094 | 3.03 | 0.082 |
|  | Year x month | 3 | 0.157 | 1.7 | 0.167 |
|  | Year x host species | 1 | 0.018 | 0.59 | 0.443 |
|  | Month x host species | 3 | 0.017 | 0.18 | 0.907 |
|  | Year x month x host species | 3 | 0.024 | 0.26 | 0.854 |
|  | Residuals | 330 | 10.188 |  |  |
| Unclassified | Year | 1 | 0.001 | 0.02 | 0.892 |
|  | Month | 3 | 0.065 | 0.35 | 0.789 |
|  | Host species | 1 | 0.134 | 2.16 | 0.143 |
|  | **Year x month** | **3** | **1.935** | **10.35** | **< 0.001** |
|  | Year x host species | 1 | 0.010 | 0.15 | 0.696 |
|  | Month x host species | 3 | 0.096 | 0.51 | 0.672 |
|  | Year x month x host species | 3 | 0.040 | 0.21 | 0.887 |
|  | Residuals | 330 | 20.561 |  |  |
| *Rhizosphere* | |  |  |  |  |
| AMF | **Year** | **1** | **0.000** | **6.35** | **0.012** |
|  | **Month** | **3** | **0.000** | **12.53** | **< 0.001** |
|  | **Host species** | **1** | **0.000** | **36.25** | **< 0.001** |
|  | **Year x month** | **3** | **0.000** | **4.63** | **0.003** |
|  | **Year x host species** | **1** | **0.000** | **14.35** | **< 0.001** |
|  | **Month x host species** | **3** | **0.000** | **8.13** | **< 0.001** |
|  | **Year x month x host species** | **3** | **0.000** | **12.53** | **< 0.001** |
|  | Residuals | 344 | 0.001 |  |  |
| ECM | Year | 1 | **0.654** | **29.1** | **< 0.001** |
|  | Month | 3 | **0.951** | **14.11** | **< 0.001** |
|  | Host species | 1 | 0.038 | 1.69 | 0.194 |
|  | Year x month | 3 | 0.116 | 1.72 | 0.163 |
|  | Year x host species | 1 | 0.013 | 0.6 | 0.44 |
|  | Month x host species | 3 | 0.063 | 0.94 | 0.423 |
|  | Year x month x host species | 3 | 0.145 | 2.16 | 0.093 |
|  | Residuals | 344 | 7.731 |  |  |
| Endophyte | **Year** | **1** | **0.011** | **29.08** | **< 0.001** |
|  | **Month** | **3** | **0.025** | **23.15** | **< 0.001** |
|  | Host species | 1 | 0.000 | 0.1 | 0.758 |
|  | **Year x month** | **3** | **0.003** | **2.99** | **0.031** |
|  | Year x host species | 1 | 0.001 | 1.54 | 0.215 |
|  | Month x host species | 3 | 0.001 | 0.81 | 0.487 |

**Table S8, continued.**

| **Guild** | **Variable** | **Df** | **Sum of sqs.** | **F** | **P** |
| --- | --- | --- | --- | --- | --- |
| *Rhizosphere* | |  |  |  |  |
| Endophyte | Year x month x host species | 3 | 0.000 | 0.13 | 0.944 |
|  | Residuals | 344 | 0.126 |  |  |
| Plant Pathogen | **Year** | **1** | **0.054** | **41.93** | **< 0.001** |
|  | **Month** | **3** | **0.063** | **16.18** | **< 0.001** |
|  | Host species | 1 | 0.000 | 0.11 | 0.735 |
|  | Year x month | 3 | 0.002 | 0.43 | 0.733 |
|  | Year x host species | 1 | 0.002 | 1.21 | 0.273 |
|  | Month x host species | 3 | 0.002 | 0.5 | 0.684 |
|  | Year x month x host species | 3 | 0.002 | 0.51 | 0.677 |
|  | Residuals | 344 | 0.444 |  |  |
| Saprotroph | **Year** | **1** | **0.071** | **11.98** | **< 0.001** |
|  | **Month** | **3** | **0.479** | **26.78** | **< 0.001** |
|  | **Host species** | **1** | **0.028** | **4.65** | **0.032** |
|  | **Year x month** | **3** | **0.115** | **6.41** | **< 0.001** |
|  | Year x host species | 1 | 0.004 | 0.65 | 0.419 |
|  | Month x host species | 3 | 0.007 | 0.38 | 0.769 |
|  | Year x month x host species | 3 | 0.035 | 1.94 | 0.123 |
|  | Residuals | 344 | 2.051 |  |  |
| Unclassified | Year | 1 | 0.006 | 0.38 | 0.536 |
|  | **Month** | **3** | **0.813** | **18.63** | **< 0.001** |
|  | Host species | 1 | 0.000 | 0.02 | 0.891 |
|  | **Year x month** | **3** | **0.170** | **3.91** | **0.009** |
|  | Year x host species | 1 | 0.002 | 0.12 | 0.733 |
|  | Month x host species | 3 | 0.094 | 2.15 | 0.093 |
|  | Year x month x host species | 3 | 0.053 | 1.22 | 0.302 |
|  | Residuals | 344 | 5.004 |  |  |
